# Modeling Stylized Facts in FX Markets with FINGAN-BiLSTM: A Deep Learning Approach to Financial Time Series

**DOI:** 10.3390/e27060635

**Published:** 2025-06-14

**Authors:** Dong-Jun Kim, Do-Hyeon Kim, Sun-Yong Choi

**Affiliations:** Department of Finance and Big Data, Gachon University, Seongnam 13120, Republic of Korea; dj010501@gachon.ac.kr (D.-J.K.); roadline5062@gachon.ac.kr (D.-H.K.)

**Keywords:** FINGAN, BiLSTM, foreign exchange, forecasting, stylized fact

## Abstract

We propose the financial generative adversarial network–bidirectional long short-term memory (FINGAN-BiLSTM) model to accurately reproduce the complex statistical properties and stylized facts, namely, heavy-tailed behavior, volatility clustering, and leverage effects observed in the log returns of the foreign exchange (FX) market. The proposed model integrates a bidirectional LSTM (BiLSTM) into the conventional FINGAN framework so that the generator, discriminator, and predictor networks simultaneously incorporate both past and future information, thereby overcoming the information loss inherent in unidirectional LSTM architectures. Experimental results, assessed using metrics such as the Kolmogorov–Smirnov statistic, demonstrate that FINGAN-BiLSTM effectively mimics the distributional and dynamic patterns of actual FX data. In particular, the model significantly reduces the maximum cumulative distribution discrepancy in assets with high standard deviations and extreme values, such as the Canadian dollar (CAD) and the Mexican Peso (MXN), while precisely replicating dynamic features like volatility clustering and leverage effects, thereby outperforming conventional models. The findings suggest that the proposed deep learning–based forecasting model holds significant promise for practical applications in financial risk assessment, derivative pricing, and portfolio optimization, and they highlight the need for further research to enhance its generalization capabilities through the integration of exogenous economic variables.

## 1. Introduction

Machine learning (ML) was introduced in 1959 by Arthur Samuel, who was working at IBM at the time, and was used to describe pattern recognition tasks that implemented learning components in pioneering artificial intelligence (AI) systems [1]. Initially, ML was considered a subset of broader AI systems, but over time, the practical applications of ML expanded significantly, surpassing the limitations defined by the AI framework [1]. The first mention of machine learning (ML) in the financial sector appeared briefly in the abstract of [2], but it was not until the study presented in [3] that ML was exclusively applied to economic problems. Due to the rapid advancement of information and database technologies, as well as significant improvements in data analysis techniques and computer hardware, the application of ML technologies has exponentially increased across various fields, including business and finance [4]. In particular, according to [5], finance is one of the most actively researched application areas for ML, showing superior performance compared to traditional models, and today ML is widely applied in financial applications. In other words, ML can handle vast amounts of structured and unstructured data more effectively, outperforming traditional economic models. The advent of ML has provided a foundation for performing financial forecasting more accurately and efficiently, thanks to its effective data processing capabilities.

Building on the rapid growth of ML in finance, recent advancements in AI have paved the way for the development of innovative generative models, particularly in the field of Generative AI. Generative AI possesses the capability to learn from existing data and generate new data, opening up innovative application possibilities across various industrial sectors. Firstly, the autoencoder (AE) compresses input data into a low-dimensional latent space and then reconstructs it back to the original data, thereby learning the important features of the data. This feature learning ability can be utilized in various applications such as noise reduction, dimensionality reduction, and data augmentation, and it holds particular potential for effectively handling complex, high-dimensional data such as financial data, making its use in the financial sector noteworthy. On the other hand, the generative adversarial network (GAN) consists of two neural networks, the Generator and the discriminator, which interact and learn together. The generator attempts to produce data that resembles real data, while the discriminator tries to distinguish whether the generated data is real or fake. Through this process, the Generator creates increasingly sophisticated data, resulting in highly realistic synthetic data. Ref. [6] first described the basic concepts and structure of GANs, presenting the principles and potential of GANs. These characteristics highlight the potential of GANs in various financial applications in various financial applications, including the simulation of financial time series data, data augmentation, and anomaly detection.

As previously mentioned, ML is currently one of the most widely used techniques in the financial sector. Although [3] was the first to apply ML to economic problems, the use of neural networks (NNs) in financial forecasting dates back to 1988, when [7] published a study predicting IBM’s daily stock returns. From the outset, ML has been primarily utilized for financial time series forecasting. Subsequently, ML has been applied to a variety of forecasting problems beyond stock return prediction alone, such as, such as forecasting Japan’s real gross domestic product (GDP) growth rate [8] and predicting credit risk [9]. In the financial sector, tasks such as classification, in addition to time series forecasting, play a very important role, and traditional time series forecasting was performed using simple point estimation methods. However, with the introduction of ML, more accurate and flexible forecasting utilizing complex time series data has become possible.

Among the recent ML-based forecasting methods, the financial generative adversarial network (FINGAN) has recently garnered significant research interest. Traditional ML and time series forecasting methodologies have certain limitations. They either cannot provide probabilistic forecasts or rely on strong distributional assumptions about future target variables, which hinders their ability to effectively reflect uncertainty [10]. According to [11], FINGAN was proposed as a deep learning-based model for financial time series data modeling. Through the training of FINGAN, it is possible to reproduce stylized facts such as heavy-tailed distributions, volatility clustering, and leverage effects, thereby effectively modeling the characteristics of financial time series data.

In this study, we propose a novel FINGAN model based on the GAN framework, specifically designed to capture the unique characteristics of financial data. To demonstrate its practical utility and effectiveness, we apply the proposed FINGAN model to generate return series in the foreign exchange market.

Building on previous work on FINGAN, this study proposes the FINGAN-BiLSTM model to more accurately capture the complex dynamics and inherent uncertainties of financial time series data. To address the limitations of conventional FINGAN, the proposed model incorporates a BiLSTM architecture, which simultaneously learns forward and backward temporal dependencies. The FINGAN-BiLSTM model comprises the following three modules: generator, discriminator, and predictor. The generator synthesizes realistic financial return sequences by combining random noise with actual time series data, while the discriminator enhances training stability by effectively distinguishing between generated and real data. Furthermore, the predictor module employs a BiLSTM framework to provide more accurate forecasting outcomes.

Recognizing the drawbacks of traditional unidirectional long short-term memory (LSTM) models, particularly the loss of contextual information due to their sequential processing, the adoption of BiLSTM becomes essential. By integrating both forward and backward contextual cues, BiLSTM provides a richer and more comprehensive representation at each time step. Financial time series data are characterized by intricate patterns, including interdependencies between past and future events, which are often reflected in stylized facts such as heavy-tailed distributions, volatility clustering, and leverage effects. The bidirectional learning mechanism of BiLSTM is expected to capture these phenomena more effectively, thereby improving both forecasting performance and overall model stability.

The proposed FINGAN-BiLSTM model is anticipated to effectively reproduce key stylized facts observed in the FX market, namely, heavy-tailed distributions, volatility clustering, and leverage effects, while also capturing financial dynamics that are often overlooked by conventional models. Moreover, this study aims to empirically demonstrate that applying the proposed model to the replication of stylized facts allows for a more robust and reliable simulation of the nuanced characteristics inherent in financial time series data.

FX return distributions deviate significantly from normality, challenging traditional financial models. Empirical studies show that FX returns exhibit heavy tails, excess kurtosis, and skewness, leading to frequent extreme fluctuations that Gaussian-based models fail to capture [12,13]. Additionally, multifractal properties and volatility clustering suggest long-range dependence in FX markets [14]. To address these issues, alternative models have been proposed, including generalized Student distributions, generalized autoregressive conditional heteroscedasticity (GARCH)-type models, and approaches based on non-extensive statistical mechanics [15,16]. These characteristics impact risk management, derivative pricing, and algorithmic trading, highlighting the need for more robust statistical models. This paper explores FX return distributions and their implications for financial modeling and forecasting.

FX market movements have long posed a significant challenge in financial research. Ref. [17] emphasized how combining multiple models can enhance prediction accuracy, while [18] introduced a directional changes framework that captures market trends beyond traditional methods. Ref. [19] explored how FX risk affects carry trade returns, and [20] investigated whether market-implied volatility holds additional predictive power. Meanwhile, ref. [21] showed that yield curves can offer valuable insights into FX returns. This paper critically reviews these forecasting strategies, highlighting their respective strengths, limitations, and potential applications in financial decision-making.

This study introduces a novel hybrid architecture, FINGAN, enhanced with BiLSTM, designed to capture the complex dynamics of FX markets. The proposed models demonstrate superior performance in replicating key stylized facts of financial time series—such as heavy-tailed, volatility clustering, and leverage effect—when benchmarked against traditional and contemporary alternatives. Notably, the incorporation of BiLSTM enhances the model’s ability to capture temporal dependencies and asymmetric information flow, resulting in more accurate and realistic estimations of the distribution of FX returns. This contributes meaningfully to the literature by offering a robust framework for distributional forecasting in FX markets, with potential implications for risk management, trading strategies, and policy modeling.

The remainder of this paper is organized as follows. Section 1 introduces the background and motivation of the study. In Section 2, we review relevant literature on the application of GAN models in finance and on FX forecasting methods. Section 3 describes the FX dataset and presents a preliminary statistical analysis of the log-return series for 12 FX pairs. Section 4 details the methodology, including both the conventional FINGAN model and the proposed FINGAN-BiLSTM architecture that incorporates BiLSTM layers. Section 5 presents the empirical results, including evaluations of the distributional goodness of fit and analyses of key stylized facts such as heavy tails, volatility clustering, and the leverage effect. In Section 6, we discuss the research findings and draw the paper’s conclusions. Finally, in Section 7, we propose directions for future research.

## 2. Literature Review

This section provides a brief review of previous studies that have applied GAN models in the field of finance, as well as existing research on FX prediction.

### 2.1. Applications of GAN Models in Financial Research

#### 2.1.1. GANin Finance

Ref. [22] utilized a GAN-based model to generate synthetic data that aligns with real financial data, aiming to reflect the upward and downward trends of the stock market and improve future stock price prediction performance. The study evaluated the generalization capability of the model by training it on various global stock indices, including Toronto Stock Exchange (TSX), Shanghai Stock Exchange (SHCOMP), Korea Composite Stock Price Index 200 (KOSPI 200), and Standard & Poor’s 500 Index (S&P 500). Experimental results demonstrated that the proposed model outperformed traditional models such as the recurrent neural network (RNN), variational autoencoder (VAE), and LSTM in terms of predictive accuracy. Notably, the model maintained high prediction accuracy during both training and testing phases, and the generated synthetic data effectively preserved the key statistical properties of real financial time series data.

Ref. [23] proposed a GAN-based framework to enhance the predictive performance of trading strategies. The study demonstrated that the discriminator in the GAN model learns more realistic trading action sequences from historical trading data, allowing it to more accurately distinguish whether a given sequence is real or generated. Additionally, experimental results showed that the GAN-based model outperformed traditional LSTM models in trading performance. The study empirically verified that GANs can exhibit superior performance in stock trading strategy prediction compared to traditional supervised learning models. In GAN-based financial market prediction, the interaction between the generator and discriminator plays a crucial role in optimizing trading strategies, ultimately enabling the development of more effective trading strategies.

Ref. [24] explored a GAN-based approach for financial scenario generation. The study proposed a conditional financial scenario generation model, integrating a bidirectional GAN (BiGAN) structure with a Markov Chain Monte Carlo (MCMC) technique under a Markov framework. This model is designed to generate multivariate financial time series independently while allowing user-defined macroeconomic scenarios to be incorporated into financial market modeling. Notably, the study highlighted that this is one of the first attempts to generate multivariate financial simulations without explicitly relying on a probabilistic model. Experimental results demonstrated that the proposed model effectively captures stylized facts of financial markets and enables realistic financial data simulation.

Ref. [25] proposed a stock price prediction model utilizing GANs and demonstrated that GANs can generate synthetic stock price data that reflects market sentiment and volatility. The study developed a model capable of effectively learning and identifying upward and downward trends in the stock market using GANs. Furthermore, the research empirically verified that GAN-based models achieve higher prediction accuracy compared to traditional statistical models (e.g., autoregressive integrated moving average (ARIMA)) and ML-based models (e.g., LSTM). Notably, the study suggested that synthetic data generated by GANs can enhance the diversity of financial datasets and serve as a supplement to improve the precision of learning from actual market data.

- Key contributions: GAN-based frameworks reliably synthesize realistic financial time series—preserving stylized facts such as heavy tails, volatility clustering, and trend dynamics—and often outperform classical approaches in predictive accuracy and data augmentation.
- Limitations: A review of the existing literature indicates that prior studies have predominantly utilized unidirectional generator and discriminator frameworks.

#### 2.1.2. PriorResearch on FINGAN Models

Ref. [11] proposed a GAN-based approach, FINGAN, for financial time series modeling. The primary objective of the study was to utilize FINGAN to generate financial time series that restore key stylized facts, such as heavy-tailed distributions, volatility clustering, and leverage effects. Additionally, the study highlighted that this was the first attempt to apply deep learning to construct a model that satisfies the fundamental statistical properties of financial time series. The experimental results demonstrated that FINGAN effectively reproduces financial time series with heavy-tailed distributions and long-range dependencies. This suggests that deep learning-based methodologies can contribute to generating more realistic financial data compared to traditional financial time series modeling approaches.

Ref. [26] described FINGAN as a model proposed to address the issue of imbalanced data in analytical customer relationship management (CRM). The model demonstrated superior area under the ROC curve (AUC) performance compared to traditional GAN models across three datasets, that is, credit card churn prediction, insurance fraud detection, and loan default prediction. These findings suggest that FINGAN is an effective approach to overcoming the challenge of imbalanced data in the financial and insurance sectors. Notably, when compared to various existing GAN models, FINGAN exhibited statistically significant performance improvements across all datasets, further enhancing its applicability in financial data analysis.

Ref. [10] conducted a study on financial time series forecasting using the FINGAN model and an economics-driven generator loss function. The results showed that FINGAN improves the Sharpe ratio, alters the generated distribution, and mitigates the mode collapse problem. Furthermore, FINGAN demonstrated superior performance in terms of the Sharpe ratio compared to ARIMA, LSTM, long-only strategies, and conventional GAN models. This suggests that FINGAN provides more sophisticated probabilistic forecasting than traditional financial time series prediction models and has strong potential for real-world applications in financial markets.

Ref. [27] conducted a study utilizing FINGAN to generate synthetic data with similar characteristics to stock market datasets. The primary objective of this research was to create artificial datasets that preserve statistical properties while preventing the disclosure of complete information from the input data. The study demonstrated that, compared to conventional GAN models, FINGAN more accurately replicates the original distribution and efficiently generates high-quality continuous synthetic financial data. Furthermore, it highlighted that this approach could help address issues related to data scarcity and limited data availability in the financial sector.

- Key contributions: The FINGAN family of GANs effectively reproduces key stylized facts of financial time series—including heavy-tailed distributions, volatility clustering, and long-range dependencies—and demonstrates superior performance compared to ARIMA, LSTM, and VAE models.
- Limitations: Prior studies have been applied primarily to stock indices and CRM/insu-rance datasets, and their generator and discriminator architectures rely on unidirectional LSTMs, precluding the modeling of bidirectional dependencies via BiLSTM.

### 2.2. Forecasting FX

#### 2.2.1. TheLiterature on FX Forecasting Using Conventional Models

Ref. [20] assessed the informational content of market-implied volatility in FX forecasting, comparing it with traditional time series models like GARCH and autoregressive moving average (ARMA). Their research evaluated whether implied volatility provides additional predictive value beyond historical data, with findings suggesting that it captures forward-looking market expectations. The study found that integrating implied volatility with traditional statistical models improves forecasting accuracy. They argued that volatility measures derived from market options enhance predictions by reflecting investor sentiment. This insight is particularly relevant for risk management strategies that rely on forward-looking volatility measures.

Ref. [28] conducted a large-scale empirical comparison of eight major machine learning models using monthly time series data from the M3 forecasting competition. Their findings showed that multilayer perceptrons (MLPs) and Gaussian processes (GPs) consistently outperformed other models in terms of predictive accuracy. The study also emphasized the critical role of preprocessing strategies—such as lagged inputs and moving averages—on forecasting performance. These insights highlight the practical suitability of MLPs and GPs for forecasting noisy and nonlinear series such as FX data, and underscore the importance of carefully selecting both models and preprocessing methods.

Ref. [21] investigated the relationship between yield curves and FX returns, arguing that interest rate differentials and term spreads can serve as reliable predictors of exchange rate movements. Their study suggests that movements in yield curves contain important signals about future currency valuations. They demonstrated that yield curve predictors improve exchange rate forecasting by incorporating macroeconomic factors into FX models. Their analysis highlights the interaction between bond markets and currency markets, revealing valuable insights for traders and policymakers. By linking yield spreads to currency expectations, they provide a foundation for macro-driven FX forecasting models.

Ref. [17] proposed a multivariate approach for FX forecasting, demonstrating how combining different models can improve prediction accuracy. They employed the principal component analysis and factor models to integrate multiple forecast sources, highlighting that diversification reduces forecasting errors. Their study emphasizes the importance of selecting appropriate weightings for combined forecasts to enhance stability. The findings suggest that integrating multiple predictive signals helps capture the inherent complexity of exchange rate movements. This approach is particularly useful for investors and policymakers seeking to minimize forecasting risk.

Ref. [19] explored the impact of FX risk on carry trade returns, showing that volatility plays a crucial role in determining future returns. Their analysis suggests that increased market uncertainty leads to the unwinding of carry trades, making volatility forecasting an essential aspect of FX prediction. They proposed that FX risk factors should be incorporated into predictive models to account for unexpected shocks. By considering risk premia and uncertainty measures, their study provides valuable insights into the predictability of currency movements. These findings help refine forecasting techniques for traders engaged in carry trade strategies.

Ref. [18] introduced a directional changes framework that identifies market turning points, providing an alternative to conventional time series models. Their method focused on capturing significant trend reversals rather than continuous price movements, offering a different perspective on market behavior. They argued that traditional models often overlook these structural changes, leading to suboptimal forecasts. By analyzing price dynamics through a directional lens, their approach improves the ability to predict short-term fluctuations. This framework enhances FX forecasting by allowing for early detection of market shifts.

Ref. [29] provided a comprehensive survey of deep learning models for time series forecasting, outlining their architectural evolution and applications. Their work particularly emphasizes the strengths of Transformer-based and attention-augmented models in capturing long-term dependencies and complex temporal patterns—features that are highly relevant to FX markets. They further discuss the use of multi-horizon forecasting, probabilistic uncertainty estimation, and the integration of static and exogenous variables. These methodological advances offer a powerful modeling toolkit for forecasting in high-frequency and high-volatility environments such as FX markets.

Ref. [30] focused on the theoretical foundations and interpretability of statistical ML approaches for time series forecasting. Regularized linear models such as the Least Absolute Shrinkage and Selection Operator (LASSO), Ridge, and Elastic Net are shown to be effective for high-dimensional forecasting tasks, particularly in selecting relevant predictors—an essential requirement in FX modeling. In addition, tree-based models and shallow NNs are presented as interpretable alternatives that balance forecasting performance with explainability. The study also advocated for ensemble and hybrid modeling strategies, which are especially useful in dealing with the structural uncertainty inherent in FX and other financial time series.

- Key contributions: Conventional models improved FX forecasting performance through implied volatility integration, MLP/GP-based preprocessing, yield curve predictors, and multivariate factor models.
- Limitations: Prior studies focused solely on point estimation based on RMSE or AUC, without modeling the full return distribution or stylized facts in financial time series.

#### 2.2.2. TheLiterature on FX Forecasting Using ML

Ref. [31] explored kernel-based methods for FX forecasting, specifically using support vector machines (SVMs) and hidden Markov models (HMMs). They introduce the concept of the Fisher kernel to enhance the predictive power of SVMs by incorporating time series dependencies. Their results show that hybrid approaches combining kernel methods and statistical models outperform standard SVM-based classifiers. The study underscores the potential of machine learning techniques in improving FX forecasting beyond traditional econometric models. Their findings highlight that kernel-based models can capture complex nonlinear patterns in FX data.

Ref. [32] proposed an artificial neural network (ANN) approach for FX forecasting, focusing on the optimization of network parameters such as the number of hidden layers, neurons, and activation functions. Their study demonstrates that properly tuned ANN models can enhance forecasting accuracy and aid in trading decision support. By leveraging historical data from the EUR/USD currency pair, they show that ANN-based models outperform conventional statistical techniques. The research emphasizes the importance of training methodology and hyperparameter selection in building effective forecasting systems. Their findings suggest that ANN models are a valuable addition to traditional FX prediction frameworks.

Ref. [33] investigated the use of deep RNNs, particularly LSTM and gated recurrent unit (GRU) networks, for FX rate prediction. Their study systematically compares these architectures against traditional feedforward networks and benchmark models. Empirical results indicate that deep networks offer superior directional forecasting accuracy, though simpler models may perform similarly in terms of profitability. Their findings emphasize the challenges of tuning deep learning models for financial forecasting. They conclude that while deep RNNs are promising, their effectiveness depends on careful architecture design and optimization.

Ref. [34] proposed a hybrid deep learning model integrating an autoencoder and LSTM networks to forecast FX volatility. The study utilizes FX Volatility Index (FXVIX) data, focusing on three major indices—Euro Volatility Index (EUVIX), British Pound Volatility Index (BPVIX), and Japanese Yen Volatility Index (JYVIX)—from 2010 to 2019. Empirical results demonstrate that the proposed autoencoder LSTM model outperforms traditional LSTM models in capturing FX volatility patterns. Additionally, the study investigates the impact of data distributions and outliers on forecasting accuracy through subperiod analysis. Their findings suggest that combining an autoencoder with the LSTM enhances the ability to learn data spread and singularities, improving volatility prediction reliability.

Ref. [35] proposed a two-layer stacked LSTM network combined with correlation analysis for FX forecasting. Their approach involves selecting datasets using the Hurst exponent to improve prediction accuracy. They compare their model with single-layer LSTM, MLP, and other NN architectures, demonstrating superior performance in terms of mean squared error (MSE) and root mean squared error (RMSE). The study highlights that integrating correlation analysis with LSTM models enhances FX prediction reliability. Their findings reinforce the potential of deep learning techniques for capturing complex relationships in currency markets.

Ref. [36] provided a systematic literature review and meta-analysis of ML approaches for FX forecasting. They analyze 60 studies covering various algorithms, including LSTM, ANN, and hybrid models. Their findings highlight that deep learning models, particularly LSTMs, have gained prominence due to their ability to capture sequential dependencies in financial data. The study also identifies key challenges such as dataset selection, model evaluation metrics, and overfitting issues. Their meta-analysis suggests that while machine learning enhances FX forecasting, further improvements are needed in feature engineering and model interpretability.

- Key contributions: Various ML methods—including kernel-based SVM/HMM, ANN, LSTM, GRU, AE-LSTM hybrids, and stacked LSTM with correlation analysis—have been shown to improve FX forecasting accuracy and directional performance.
- Limitations: These approaches mainly focus on point-estimate metrics such as MSE, RMSE, and directional accuracy. In other words, existing ML-based FX forecasting studies do not model the full return distribution or the stylized facts of financial time series.

In this study, we propose a novel FIN-GAN-based model by integrating a BiLSTM architecture into the traditional FINGAN framework. This new hybrid model is designed to generate synthetic data that closely resembles real FX return data. Our research contributes to the growing body of literature on the application of GANs in finance by introducing a new model architecture. Furthermore, by demonstrating its predictive performance in the context of FX markets—one of the core assets in the global financial system—our study also offers meaningful practical implications.

## 3. Data Description

The FX data used in this study comprise exchange rate information for six major and six minor currencies based on trading volume. Data were retrieved from the Yahoo Finance platform covering the period from 1 January 2020, to 31 December 2024. Descriptive statistics were computed based on the log returns of each dataset, serving as the foundation for the subsequent analysis. The study deliberately employs post-2020 data to accurately capture the structural shifts in the market following the COVID-19 pandemic and to mitigate discrepancies associated with pre-pandemic data.

Table 1 provides the basic descriptive statistics for the daily log returns of 12 major and minor currency pairs, such as the Euro (EUR), Japanese yen (JPY), British pound (GBP), Australian dollar (AUD), Canadian dollar (CAD), Swiss franc (CHF), Brazilian real (BRL), Korean won (KRW), Mexican peso (MXN), Singapore dollar (SGD), South African rand (ZAR), and Chinese yuan (CNY). Overall, the average returns are exceedingly close to zero. For example, the EUR exhibits an average return of −0.0001, the JPY −0.0003, and the GBP approximately zero. Although the differences are subtle, these figures suggest that each currency pair displays distinct return characteristics in response to the evolving market and economic conditions.

A detailed examination of the extreme values reveals substantial heterogeneity among the currencies. The ZAR, for instance, reaches a maximum return of 0.2041 and drops to a minimum return of negative 0.2009, indicating a pronounced potential for extreme fluctuations. Similarly, the BRL demonstrates a considerable range by attaining a maximum return of 0.0907 and a minimum return of −0.0863. In contrast, the SGD is characterized by a more constrained behavior with its maximum return at 0.0129 and its minimum return at −0.0150, pointing to a comparatively stable return profile. The CAD and the CHF also display unique distribution characteristics; the CAD attains a maximum return of 0.0319 and a minimum return of −0.0297, while the CHF reaches a maximum return of 0.0284 and a minimum return of −0.0226.

Volatility, as measured by the Std. dev., further accentuates these differences. The ZAR and the BRL, with Std. dev. of 0.0123 and 0.0111, respectively, indicate higher volatility, whereas the SGD, with a standard deviation of only 0.0029, reflects a notably lower level of fluctuation. In addition, measures of skew and kurt offer further insight into the asymmetry and tail behavior of the return distributions. For example, the ZAR exhibits a skew of 0.0616 along with a kurt of 111.1518, indicating extreme deviation from normality. The BRL similarly deviates from normality, with a skew of 0.0834 and a kurt of 7.6854. Additional evidence of non-normality is observed in the CAD, which shows a skew of −0.0167 and a kurt of 4.3769, in the CHF, which has a skew of 0.2965 and a kurt of 2.4943, and in the CNY, which records a skew of 0.3246 and a kurt of 7.7546.

These distributional characteristics are further substantiated by the J.-B. test statistics. The ZAR records a test statistic of 665,572.10, while the BRL exhibits a value of 3179.91. Likewise, the CAD and the CHF display significant test statistics of 1030.01 and 352.98, respectively, indicating marked departures from normality. Furthermore, the outcomes of the ADF test and the PP test provide additional support for the stationarity of the daily log returns across these currency pairs.

In summary, although the daily log returns of these 12 major and minor currency pairs are characterized by average values that approximate zero, there are significant differences in terms of extreme returns, volatility, skew, kurt, and normality as indicated by the J.-B. test. Notably, the ZAR and the BRL are distinguished by their high volatility and an increased propensity for experiencing extreme return values.

**Figure 1:**
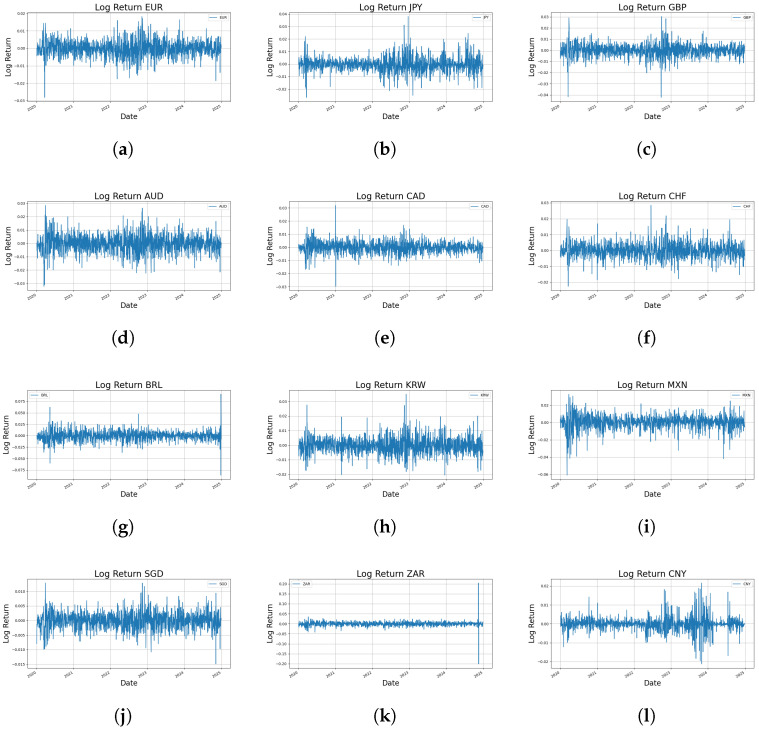
Log return for each ticker from 1 January 2020 to 31 December 2024. (**a**) EUR, (**b**) JPY, (**c**) GBP, (**d**) AUD, (**e**) CAD, (**f**) CHF, (**g**) BRL, (**h**) KRW, (**i**) MXN, (**j**) SGD, (**k**) ZAR, (**l**) CNY.

Figure 1 presents the time series patterns of daily log returns for each currency pair, illustrating how the statistical properties—such as mean, variance, extreme values, skewness, and kurtosis—summarized in Table 1 manifest over time.

In this section, we provide a detailed analysis of the time series patterns for each currency to elucidate the dynamic characteristics and inherent risk factors observed in the FX market.

The ZAR exhibits high volatility, with extreme outliers, particularly in late 2024. This behavior is consistent with its wide return range and exceptionally high kurtosis (111.15). Similarly, the BRL showed major fluctuations in mid-2020, late 2022, and 2024, reflecting high risk associated with Brazil’s economic instability and sensitivity to global commodity prices.

In contrast, the SGD displays notable stability, with narrow return bounds and low Std. dev. (0.0029), possibly attributable to effective financial management. The EUR shows moderate volatility, with a sharp decline in early 2020, likely linked to economic or policy shocks within the eurozone. This indicates that, within the 2020 study period, following the pronounced downturn in early 2020, a moderate level of volatility persisted in subsequent intervals, as evidenced by Figure 1.

The CAD and CHF are relatively stable but reveal short-lived volatility spikes (CAD in 2021, CHF in early 2020 and mid-2022). The GBP and JPY indicate sensitivity to short-term shocks; GBP exhibits pronounced fluctuations and high kurtosis (6.01), while JPY remains generally stable.

KRW and MXN show moderate volatility, with visible volatility clustering in early 2020 and late 2022 for the KRW and frequent fluctuations (MXN), reflecting their responsiveness to market uncertainty. The AUD maintains moderate, steady volatility, while CNY remains stable overall, with a discernible rise in volatility during 2023–2024 amid heightened global uncertainty.

Overall, the time series patterns in Figure 1 underscore the distinct dynamics of each currency, reflecting stylized facts such as non-normality, volatility clustering, and extreme events—thereby providing essential insights into FX market behavior.

## 4. Methods

### 4.1. FINGAN

In recent years, a FINGAN-based model has been proposed to effectively replicate the uncertainties and intricate statistical properties inherent in financial time series data. Traditional ML and time series forecasting techniques are limited in that they either fail to provide probabilistic forecasts or require overly stringent assumptions regarding the distribution of future values, thereby inadequately capturing the inherent variability and uncertainty of empirical data [10]. In response, ref. [11] introduced a deep learning–based FINGAN model, which has been empirically shown to effectively reproduce stylized facts such as heavy-tailed distributions, volatility clustering, and the leverage effect.

The FINGAN framework fundamentally relies on an adversarial training mechanism between a generator and a discriminator. The generator synthesizes financial time series data that mimics real data by integrating a random noise vector with salient features derived from empirical observations, while the discriminator is aimed at distinguishing between real and generated data. Through this adversarial process, both networks iteratively refine their performance, ultimately enabling the generator to approximate the complex distribution of actual financial data with remarkable precision.

### 4.2. FINGAN with BiLSTM

In this study, we propose the FINGAN-BiLSTM model, an extension of the conventional FINGAN model. The proposed model incorporates a BiLSTM architecture across the generator, discriminator, and predictor networks. This integration overcomes the limitations of unidirectional LSTM-based models and enables more effective learning of the complex temporal patterns inherent in financial time series data.

Within the generator network, the input sequence is concatenated with a random noise vector and processed through two successive BiLSTM layers to learn bidirectional temporal dependencies. Subsequently, layer normalization and Gaussian Noise are applied. The output then passes through a residual connection and a dense layer, where tanh activation and batch normalization are employed to generate synthetic financial time series data that closely resemble the real data.

The discriminator network employs BiLSTM layers to extract the temporal characteristics of the input sequence effectively. Following feature extraction, the resultant feature vector is flattened and processed through a dense layer, where it is combined with the target data to effectively distinguish between the generated and real data.

In the predictor network, BiLSTM layers are utilized to capture the intricate patterns and volatility inherent in financial time series data. The input sequence is first processed through a BiLSTM layer to acquire bidirectional temporal dependencies, after which the output is transformed into a one-dimensional vector via a flattening layer. This vector is then passed through sequential dense and dropout layers to yield the final prediction, which serves as the basis for replicating stylized facts such as volatility clustering and heavy-tailed distribution.

During training, an L2 loss-based GAN (LSGAN) loss function is applied to both the generator and discriminator networks. Additionally, an L1 loss (absolute error) is applied on the predictor network to minimize the discrepancy between the real and predicted values. The training process utilizes mini-batch learning via Gradient Tape, and a Loss Scale Optimizer is employed to perform gradient scaling and clipping associated with mixed precision training, thereby ensuring training stability.

Hyperparameters were optimized using a grid search over candidate combinations, with the optimal configuration selected based on the RMSE of the predictor network during the initial tuning phase. The model’s predictive performance is evaluated using traditional metrics such as RMSE, MSE, mean absolute error (MAE), R^2^, and mean absolute percentage error (MAPE), along with the Kolmogorov–Smirnov statistic (KS_Stat) to quantitatively assess the differences between distributions. Moreover, various visualization tools, including quantile–quantile (QQ) plots, heavy-tailed analysis, volatility clustering, and the leverage effect, are employed to thoroughly examine the degree to which the predicted data reproduces the distributional and temporal characteristics of the real data. Evaluation results demonstrate that the proposed FINGAN-BiLSTM model accurately reproduces stylized facts inherent in financial time series data, including heavy-tailed distributions, volatility clustering, and the leverage effect.

### 4.3. Model Architecture and Settings

**Figure 2:**
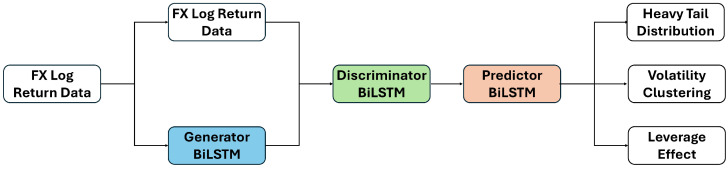
Overview of FINGAN-BiLSTM.

**Figure 3:**
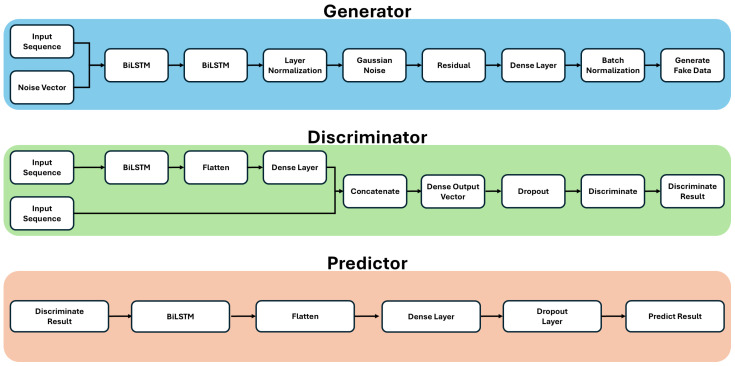
Architecture of each component of the FINGAN-BiLSTM.

Figure 2 presents the overall structure of the FINGAN-BiLSTM model, while Figure 3 details the internal architecture of each generator, discriminator, and predictor module. The main hyperparameters and training configurations for these components are summarized in Table 2. Here, “G” denotes the generator network, “D” the discriminator network, and “P” the predictor network. Sequence length *L*, input scaling, and batch size were held constant across all experiments. The noise dimension and BiLSTM depth in the generator were tuned to enhance diversity while preserving temporal context, whereas discriminator and predictor dropout rates were set low to maintain adversarial balance and forecasting robustness. Training was performed using Adam optimizers with separate learning rates and moment coefficients ($\beta_{1}$, $\beta_{2}$) for G, D, and P, with AMSGrad enabled for the generator. Gradient clipping (norm = 1.0) was applied to prevent exploding gradients, and regularization weights for auxiliary loss, drift penalty, and gradient penalty were selected based on a preliminary RMSE-guided grid search to ensure stable convergence and faithful reproduction of FX return stylized facts.

### 4.4. Grid-Search-Based Hyperparameter Tuning

Hyperparameter tuning for the proposed FINGAN-BiLSTM model was performed using a comprehensive grid search approach aimed at maximizing its performance. This process entails a systematic exploration of all possible combinations within a predefined set of candidate hyperparameter values, thereby quantitatively evaluating the model performance for each configuration to identify the optimal parameter set. Specifically, candidate values were defined for key hyperparameters such as the number of BiLSTM units, learning rate, dropout rate, and batch size for generator, discriminator, and predictor models, as well as for auxiliary parameters including the auxiliary loss coefficient and drift coefficient. The auxiliary loss coefficient functions as a weighting factor for an additional loss term computed within the generator network to ensure that the generated data more effectively reflects the characteristics of the real data, while the drift coefficient is applied to a regularization term during GAN training to mitigate excessive fluctuations in the discriminator output, thereby enhancing overall training stability.

All candidate combinations were generated via the Cartesian product, and each configuration was evaluated using a designated training and validation dataset. The performance of the predictor was quantified through the calculation of the RMSE, which precisely measures the deviation between the predicted and actual values at each data point, thus facilitating the selection of the optimal hyperparameter configuration.

Although the final performance evaluation and comparisons with alternative models employed the KS_Stat to assess the goodness of fit between predicted and actual distributions, RMSE was prioritized during the hyperparameter tuning phase due to its capability to directly capture the magnitude of prediction errors. Moreover, hyperparameter tuning was performed separately for each FX to derive a customized configuration that reflects the unique characteristics and data distributions of each asset. Ultimately, the optimal hyperparameter configuration obtained through this process is expected to enhance the overall predictive accuracy of the model and effectively capture the complex dynamics inherent in financial time series data.

## 5. Empirical Results

In this section, we present the prediction results for the 12 FX return series using the FINGAN-BiLSTM model. First, we generate synthetic return distributions and compare them with the corresponding real return distributions. Subsequently, we evaluate the quality of the synthetic data by examining its adherence to key stylized facts commonly observed in financial time series, namely heavy-tailed, volatility clustering, and leverage effects.

For clarity, all metrics and diagnostic plots in this section—such as the KS_Stat, tail exponent α, volatility decay exponent β, and leverage effect L(k)—are computed on a single synthetic return trajectory generated by the model.

### 5.1. Return Distribution

In this study, the Kolmogorov–Smirnov statistic, denoted as KS_Stat, is employed to quantitatively assess the difference between the distribution of predicted values and that of observed values. The KS_Stat is a nonparametric measure that quantifies the maximum absolute difference between two cumulative distribution functions (CDFs). The KS_Stat is defined as follows (Equation (1)):

(1) $$D_{n,m}=\underset{x}{\sup}\left|F_{n}\left(x\right)-G_{m}\left(x\right)\right|,$$

where

(2) $$F_{n}\left(x\right)=\frac{1}{n}\sum_{i=1}^{n}1\left\{X_{i}\leq x\right\}$$

represents the empirical distribution function (EDF) of the predicted values, and

(3) $$G_{m}\left(x\right)=\frac{1}{m}\sum_{j=1}^{m}1\left\{Y_{j}\leq x\right\}$$

denotes the EDF of the observed values.

In the ideal case where the predicted and observed distributions are identical, the KS_Stat converges to zero, whereas larger discrepancies between the distributions result in an increased value of $D_{n,m}$. Moreover, the KS test does not require any assumptions regarding the underlying distributional forms, thereby providing robust applicability to a wide range of distributional shapes.

Table 3 presents the KS_Stat as the primary metric for assessing how well the distribution predicted by the FINGAN-BiLSTM model reflects the actual data distribution. The model has demonstrated its capacity to capture key statistical features—such as tail behavior and the occurrence of extreme values—as evidenced by low KS_Stat values for many FX tickers. For instance, for EUR, AUD, and CAD, the KS_Stat values computed by the FINGAN-BiLSTM model are 0.0610, 0.0650, and 0.0894, respectively. The EUR exhibits moderate volatility (Std. dev. = 0.0047) and occasional policy-driven shocks, the AUD shows relatively stable fluctuations (Std. dev. = 0.0068), and the CAD—with a higher Std. dev. of 0.0044 and wider extreme-value range—poses a greater challenge for distributional emulation. Nonetheless, the proposed model successfully minimizes the maximum divergence between predicted and empirical cumulative distribution functions (ECDFs) by effectively learning complex distributional shapes and high-volatility behavior. This finding suggests that the integration of the GAN-based augmentation module with a BiLSTM enables precise emulation of distributional properties, including significant tail risk and extreme movements.

In contrast, in the case of CNY, prior studies have reported that external factors such as aggressive market interventions and policy adjustments by the central bank significantly influence the FX return distribution [37,38]. These external factors entail complex nonlinearities that are difficult to capture solely by conventional statistical characteristics, and because the FINGAN-BiLSTM model is primarily trained on historical data, it has limitations in fully reflecting abrupt market shifts such as policy changes or central bank interventions. Consequently, for CNY, the predicted distribution fails to effectively reproduce the abnormal variations or structural changes observed in the actual distribution, resulting in a relatively high KS_Stat.

Thus, the FINGAN-BiLSTM model effectively minimizes discrepancies between predicted and actual distributions for tickers with moderate to high volatility—such as EUR (0.0610), AUD (0.0650), and CAD (0.0894)—while yielding a relatively higher KS_Stat for CNY, where external interventions play a critical role.

**Figure 4:**
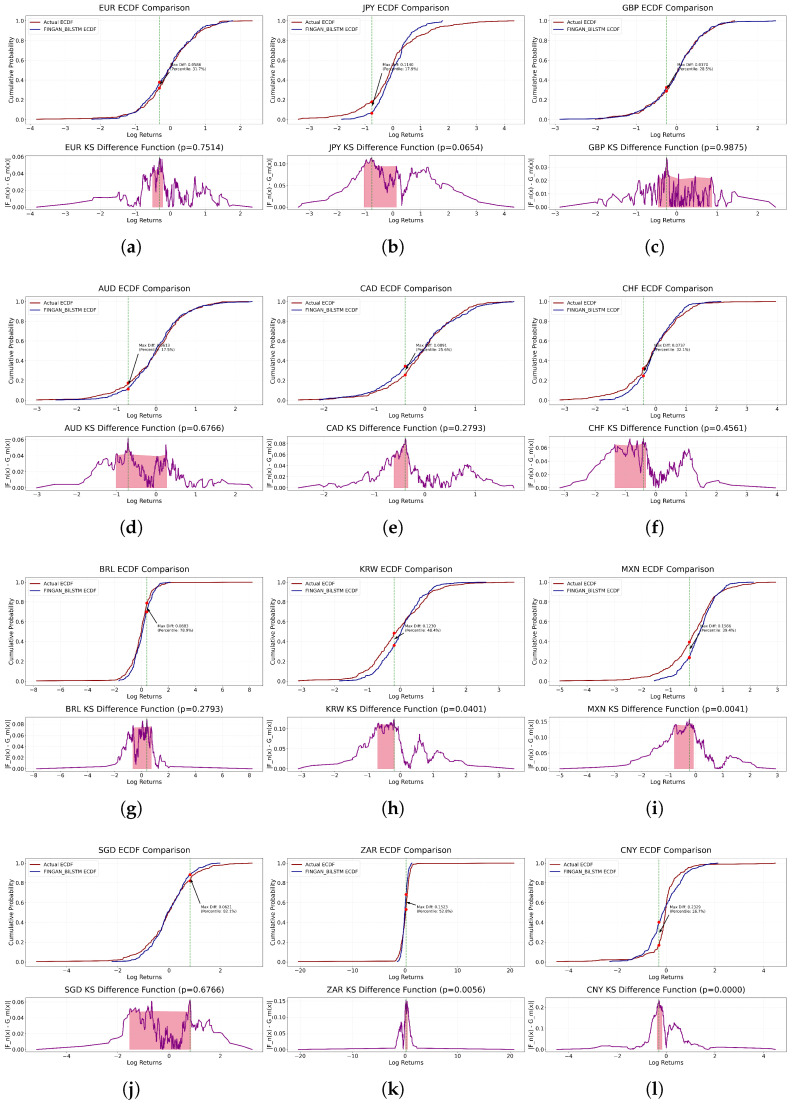
Kolmogorov–Smirnov Statistics of the Predicted Time Series Generated by the FINGAN-BiLSTM Model. (**a**) EUR, (**b**) JPY, (**c**) GBP, (**d**) AUD, (**e**) CAD, (**f**) CHF, (**g**) BRL, (**h**) KRW, (**i**) MXN, (**j**) SGD, (**k**) ZAR, (**l**) CNY. The green dashed line marks the log-return of maximal KS distance; the red shading indicates $|F_{n}\left(x\right)-G_{m}\left(x\right)|$ beyond the 90th percentile.

Figure 4 visualizes the results of Table 3, depicting the maximum deviation between the empirical log-return distribution and the synthetic time series generated by the FINGAN-BiLSTM model. Overall, most currency pairs exhibit *p*-values exceeding 0.05, indicating that the predicted distributions approximate normality. However, CNY constitutes an exception, for which a statistically significant deviation (*p* < 0.0001) is observed. This anomaly is consistent with prior findings [37,38], whereby aggressive central-bank interventions and policy adjustments impart strong nonlinear effects on the FX return distribution, thereby undermining the attainability of conventional significance thresholds.

Among the developed-market currencies, GBP (KS_Stat = 0.0370, *p* = 0.9875), EUR (KS_Stat = 0.0586, *p* = 0.7514), AUD (KS_Stat = 0.0613, *p* = 0.6766), and SGD (KS_Stat = 0.0621, *p* = 0.6766) demonstrate superior goodness-of-fit. Their low KS_Stat and high *p*-values indicate that the synthetic and empirical distributions do not differ in a statistically significant manner. In particular, despite the maximum deviation occurring in the mid-percentile range for GBP and EUR, the model faithfully reproduces the overall distributional shape. Similarly, AUD and SGD appear to capture moderate-range volatility dynamics more precisely than extreme-value behavior.

In contrast, emerging-market currencies such as CNY (KS_Stat = 0.2329, *p* < 0.0001) and ZAR (KS_Stat = 0.1523, *p* = 0.0056) exhibit significant distributional mismatches. CNY’s maximum deviation arises in the tail region, indicating insufficient modeling of extreme movements. ZAR’s largest discrepancies occur within the negative-return quantiles, suggesting that structural regime shifts or sudden market interventions were not fully incorporated.

Intermediate performance is observed for CAD (KS_Stat = 0.0891, *p* = 0.2793), CHF (KS_Stat = 0.0737, *p* = 0.4561), JPY KS_Stat = (0.1140, *p* = 0.0654), and KRW KS_Stat = (0.1230, *p* = 0.0401). These currencies also record their maximum KS deviations within central percentiles. Notably, KRW’s *p*-value of 0.0401 approaches the conventional significance threshold, indicating potential scope for further model refinement.

In summary, while the FINGAN-BiLSTM model demonstrates robust capability in reproducing the central tendency and moderate-range volatility of developed-market FX pairs, it remains challenged by the frequent extreme-value occurrences and structural regime changes characteristic of emerging-market currencies.

**Figure 5:**
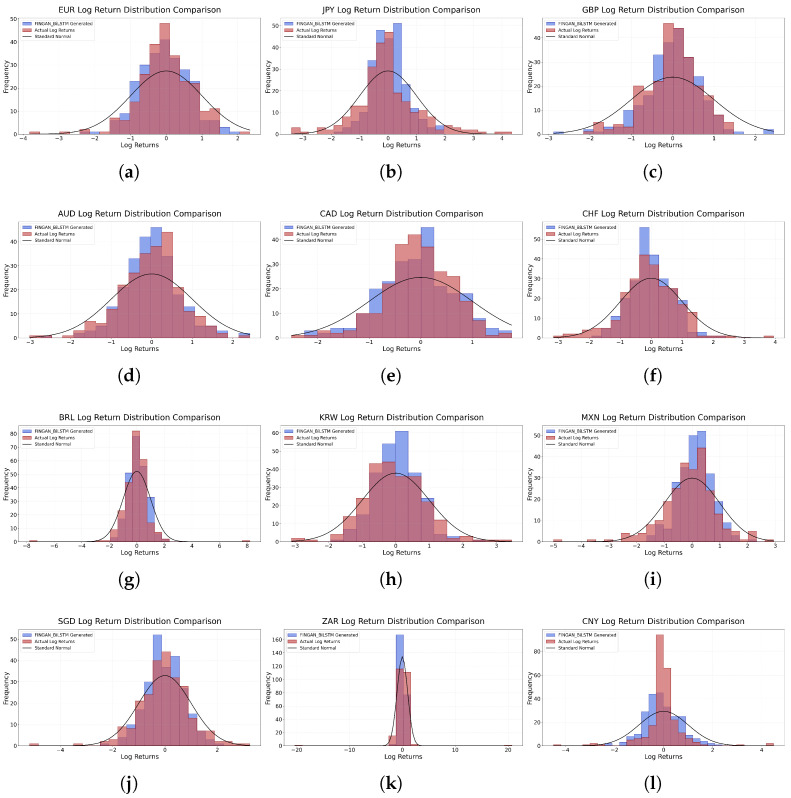
Histogram comparing the log-return distribution predicted by the FINGAN-BiLSTM model with the empirical log-return distribution. (**a**) EUR, (**b**) JPY, (**c**) GBP, (**d**) AUD, (**e**) CAD, (**f**) CHF, (**g**) BRL, (**h**) KRW, (**i**) MXN, (**j**) SGD, (**k**) ZAR, (**l**) CNY.

**Figure 6:**
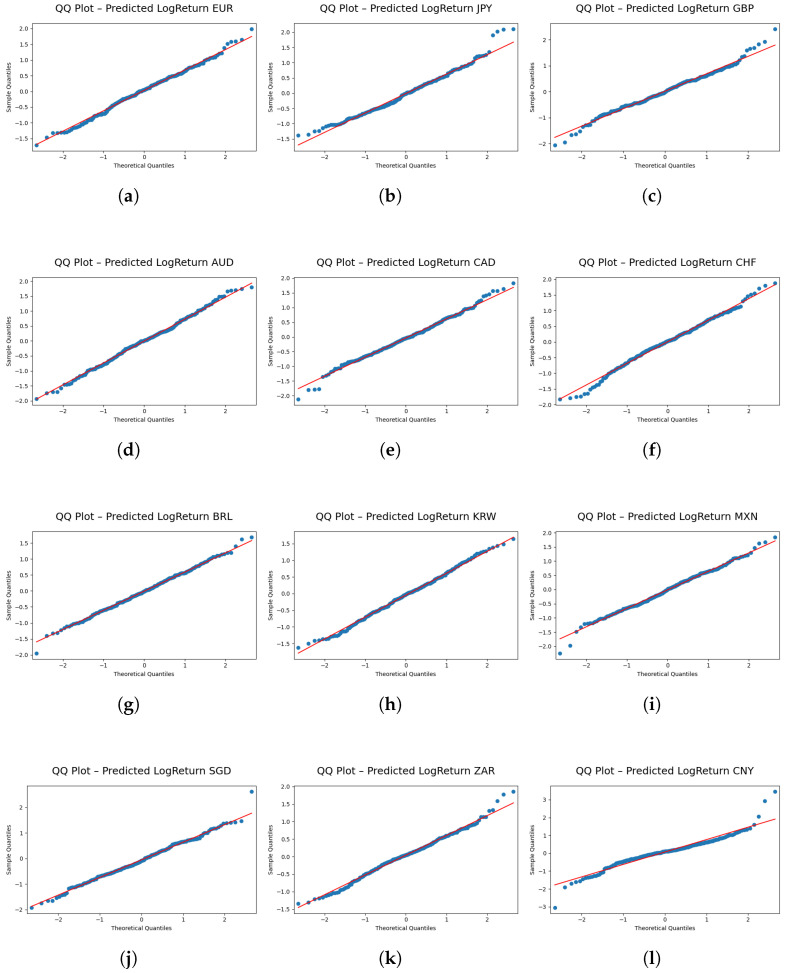
QQ plot predicted by FINGAN-BiLSTM. (**a**) EUR, (**b**) JPY, (**c**) GBP, (**d**) AUD, (**e**) CAD, (**f**) CHF, (**g**) BRL, (**h**) KRW, (**i**) MXN, (**j**) SGD, (**k**) ZAR, (**l**) CNY. The blue dots represent the empirical quantiles of the predicted log returns, while the red dashed line indicates the 45-degree reference line, where the empirical and theoretical quantiles would align under a perfect fit.

Figure 5 and Figure 6 serve as visual diagnostic tools for evaluating the fidelity of the predicted return distributions.

Figure 5 illustrates the log-return distributions generated by the FINGAN-BiLSTM model. This figure visually demonstrates the extent to which the model effectively captures the heavy-tailed properties and the occurrence of extreme values, the asymmetry, and the tail behavior of financial data. The figure is designed to provide an overview of the prediction results for each asset, with the understanding that direct comparisons with the empirical distribution should be supported by the numerical evaluations provided in Table 3. Furthermore, the visual comparison with the empirical distribution, complemented by the numerical evaluations presented in Table 3, facilitates an intuitive assessment of the predictive accuracy of the FINGAN-BiLSTM model. Moreover, this visual representation serves as a diagnostic tool for assessing how well the predicted distributions reflect structural imbalances and non-standard market features. Notably, the figure indirectly indicates that the model exhibits robust performance for certain assets, such as GBP and EUR, while showing limitations for assets that are particularly sensitive to exogenous influences, such as CNY.

Figure 6 provides a QQ plot comparing the quantiles of the predicted log-return distribution with those of an ideal reference distribution. This diagnostic plot is a critical tool for visually assessing the fidelity with which the model replicates the tail characteristics of the actual data. The QQ plot, when considered in conjunction with the numerical assessments in Table 3, facilitates a comprehensive evaluation of the FINGAN-BiLSTM model’s performance in capturing the distributional properties of financial time series data.

Nevertheless, a closer inspection of Figure 6 reveals systematic under-fitting in the left tail for certain currency pairs, most prominently ZAR and BRL. In these cases, the model’s predicted quantiles fall below the empirical quantiles, indicating that extreme negative returns are underestimated. Because risk measures such as value-at-risk (VaR) and expected shortfall (ES) are highly sensitive to tail behavior, this underestimation may lead to overly optimistic assessments of downside risk.

### 5.2. Stylized Facts

While canonical empirical studies often report tail exponents α in the interval (3,5) [39,40], this benchmark reflects aggregate findings across diverse assets and market regimes and should not be interpreted as a strict acceptance criterion for individual FX pairs. Market structure, central-bank interventions, and regime shifts may induce true tail behavior that falls outside this range. Therefore, in the present study, we employ (3,5) merely as a contextual reference.

Table 4 reports the empirically estimated tail exponents (α) and volatility decay exponents (β) for each of the twelve selected FX pairs. The tail exponent α varies from 2.5402 (MXN) to 5.5990 (CAD), indicating differing degrees of heavy-tailed behavior across currency markets. Likewise, the decay exponent β spans from −0.1342 (CHF) to 3.4440 (ZAR), reflecting heterogeneity in the persistence of volatility. These benchmark values provide a reference for evaluating the ability of our FINGAN-BiLSTM model to replicate the stylized distributional properties observed in real FX log-return data.

#### 5.2.1. Heavy-TailedDistribution

In financial time series analysis, extreme events occur with a significantly higher probability than that predicted by a normal distribution. In financial time series data, the heavy-tailed characteristic signifies that the probability of extreme events occurring is substantially higher than that predicted by a normal distribution [41,42]. This phenomenon is a crucial element for quantifying the extreme distribution of asset price fluctuations, thereby holding considerable significance for risk management and derivative pricing. In this study, the heavy-tailed characteristic is analyzed through two principal components. First, the probability density function (PDF) is expressed as follows:

(4) $$f\left(x\right)=c\cdot x^{-\gamma }$$

In Equation (4), *c* is the normalization constant ensuring that the total probability is unity, and γ>0 is the parameter that determines the rate of tail decay. A smaller value of γ implies a slower decay of the tail, which in turn increases the relative likelihood of observing extreme values. Another important representation is the complementary cumulative distribution function (CCDF), given by

(5) $$S\left(x\right)=P\left(X\geq x\right)\propto x^{-\alpha }$$

In Equation (5), α denotes the tail exponent; a smaller value of α indicates a heavier tail of the distribution. Empirical observations in financial markets typically report that the tail index α assumes values within the range of 3 to 5 [39,40]. This finding suggests that extreme events occur far more frequently than would be expected under a normal distribution. Thus, the heavy-tailed property amplifies the likelihood of extreme events in financial markets [42,43]. It further implies that asset price fluctuations do not follow an independent and identically distributed (i.i.d.) process but rather exhibit clusters of high volatility over specific intervals. Moreover, the persistence of high volatility states indicates a long-term temporal dependence that is not readily captured by conventional normal distribution models or white noise assumptions. In this study, the complementary cumulative distribution function (CCDF) form was fitted to the normalized positive returns of FX data using the powerlaw package and estimation methods such as the maximum likelihood estimation and nonlinear least squares, thereby estimating the tail exponent α. In addition, the decay parameter γ of the PDF was analyzed to quantitatively assess how effectively the return distributions of various currency pairs capture extreme volatility.

Therefore, the heavy-tailed characteristic increases the probability of extreme events in financial markets and functions as an important risk factor that investors and risk managers must consider. This property serves as a critical benchmark for models seeking to replicate the complex risk characteristics observed in real markets, particularly in terms of accurately assessing loss risks under extreme conditions.

**Figure 7:**
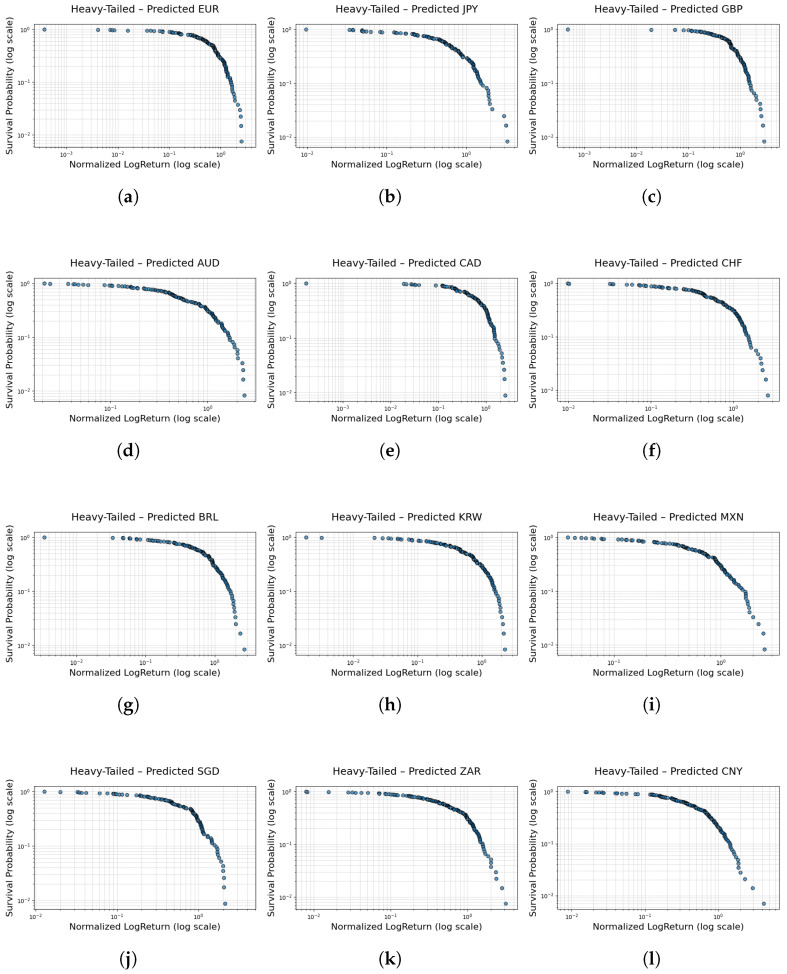
Heavy-tailed distribution predicted by FINGAN-BiLSTM. (**a**) EUR, (**b**) JPY, (**c**) GBP, (**d**) AUD, (**e**) CAD, (**f**) CHF, (**g**) BRL, (**h**) KRW, (**i**) MXN, (**j**) SGD, (**k**) ZAR, (**l**) CNY.

Figure 7 and Table 5 present results demonstrating that the FINGAN-BiLSTM model effectively reproduces the heavy-tailed characteristics observed in real FX exchange rate data for most cases. For instance, in the case of EUR, the tail index α predicted by the FINGAN-BiLSTM model is 4.4033, which falls within the range of values typically encountered in financial time series, while the predicted values for JPY and GBP are 4.0506 and 4.0041, respectively. These results suggest that the model helps to alleviate the underestimation of extreme event probabilities inherent in traditional normal distribution models.

In particular, compared to standalone LSTM or BiLSTM models, the FINGAN-based model demonstrates an improved ability to reproduce realistic heavy-tailed characteristics. For example, in the case of CAD, the LSTM model produced an excessively high tail index (α=355.7515), whereas the FINGAN-BiLSTM model yielded a value of 3.4677, thereby more appropriately reflecting the distributional properties of actual financial data. Furthermore, even in cases where the data is significantly affected by outliers, such as MXN, the FINGAN-BiLSTM model produces robust tail index estimates, demonstrating enhanced resistance to outliers.

However, the predictive performance of the FINGAN-BiLSTM model is not uniformly superior across all FX exchange rates. For example, in the case of ZAR, as indicated by the basic statistics in Table 1, although the mean is near zero, the Std. dev. (0.0123), extreme values (maximum 0.2041, minimum −0.2009), and an exceptionally high kurt (111.1518) reveal that the distribution is highly abnormal. These characteristics are also clearly observed in the log-return distribution (Figure 1), where the ZAR data exhibit frequent outliers and high volatility. Due to these data characteristics, the FINGAN-BiLSTM model encountered difficulties in generating a stable distribution during training, resulting in a failure to fully capture the heavy-tailed properties. Specifically, for ZAR, the predicted tail index is 2.9069, which falls short of the generally expected value of 3 or higher. This outcome provides evidence that the extreme values and high volatility of the ZAR data adversely affected the model’s learning process.

#### 5.2.2. VolatilityClustering

Volatility clustering is one of the key stylized facts in financial time series data. It refers to the phenomenon wherein asset price fluctuations are not randomly distributed but instead exhibit clusters of high volatility over certain periods [44,45]. The tendency for high-volatility states to persist consecutively indicates a long-term temporal dependence that is not readily captured by conventional models assuming homoskedastic, white-noise residuals [46,47]. However, even under the assumption of normally distributed innovations, approaches such as GARCH-type models—which explicitly model time-varying variance—can effectively capture volatility persistence.

In this study, based on the asset’s logarithmic returns r(t), the autocorrelation function of the absolute returns |r(t)| was utilized to quantitatively evaluate volatility clustering. Specifically, for each time point in the series {r(t)}, the absolute value |r(t)| was computed, and the autocorrelation coefficient (ACF) for a lag *k* was calculated as follows:

(6) $$\mathrm{ACF}\left(k\right)=\frac{\sum_{t=1}^{N-k}\left(\left|r\left(t\right)\right|-\bar{\left|r\right|}\right)\left(\left|r\left(t+k\right)\right|-\bar{\left|r\right|}\right)}{\sum_{t=1}^{N}{\left(\left|r\left(t\right)\right|-\bar{\left|r\right|}\right)}^{2}}$$

In Equation (6), $\bar{\left|r\right|}$ denotes the mean of the absolute returns over the entire time series. This equation measures the correlation between absolute returns at various lags, thereby aiding in the assessment of how persistently high volatility is maintained over time.

Furthermore, to elucidate the decay pattern of the observed autocorrelation coefficients, these values were fitted to a power-law form:

(7) $$\mathrm{ACF}\left(k\right)=a\cdot k^{-\beta }$$

In Equation (7), *a* is a fitting constant and β is the exponent that characterizes the decay rate of the autocorrelation with respect to the lag *k*. In general, if β>0, the autocorrelation coefficient decreases gradually with increasing lag; a smaller value of β indicates that high volatility tends to persist for an extended period. This serves as an important indicator that extreme price fluctuations in financial markets are not merely random or isolated occurrences but are instead observed in consecutive intervals.

**Figure 8:**
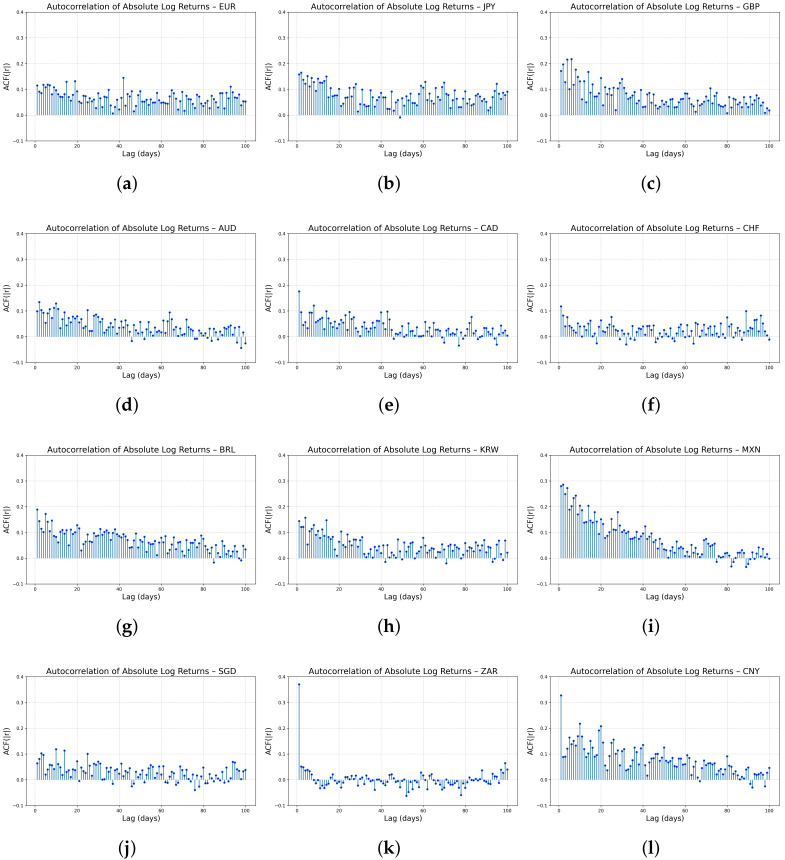
Empirical ACF of absolute log returns |r(t)| for 12 FX pairs, up to lag 100. (**a**) EUR, (**b**) JPY, (**c**) GBP, (**d**) AUD, (**e**) CAD, (**f**) CHF, (**g**) BRL, (**h**) KRW, (**i**) MXN, (**j**) SGD, (**k**) ZAR, (**l**) CNY.

Equation (7) summarizes the decay of volatility clustering through the exponent β. As Figure 8 shows, microstructure noise further suppresses autocorrelation in the short horizon (k<3) [48,49], whereas in the long horizon (k>50) volatility has largely reverted to its mean, flattening the ACF near zero [50]. Consequently, a pure power-law relationship holds only for 3≤k≤30. When the analysis is restricted to this lag window, Equation (7) remains a valid model for describing volatility clustering in FX markets.

Volatility clustering is interpreted as arising from a variety of factors, including the information processing behavior of market participants, investor sentiment, and underlying structural factors. For example, when an economic event or policy announcement induces an information shock that spreads throughout the market, investors may adopt conservative strategies in response to uncertainty, resulting in prolonged periods of high volatility. Such dynamics are crucial for both short-term risk pricing and long-term portfolio management. Through this analysis, the study evaluates the effectiveness of the model in replicating the complex phenomenon of volatility clustering observed in real financial markets, thereby exploring potential enhancements in financial risk assessment models.

These empirically estimated decay exponents β provide a quantitative reinforcement of the qualitative volatility patterns described in Section 3. Specifically, the low values of β for CAD (0.0252) and CHF (–0.0751) formally capture the rapid dissipation of volatility following the short-lived spikes observed in CAD during 2021 and in CHF during early 2020 and mid-2022. Likewise, the modestly positive β for GBP (0.0018), in contrast to the near-zero β for JPY (0.0279), faithfully reflects GBP’s heightened sensitivity to short-term shocks and JPY’s relative stability as noted in Section 3. Thus, the power-law fit of the ACF over the 2020 sample serves as a consistent, quantitative counterpart to the visual patterns highlighted in Section 3.

**Figure 9:**
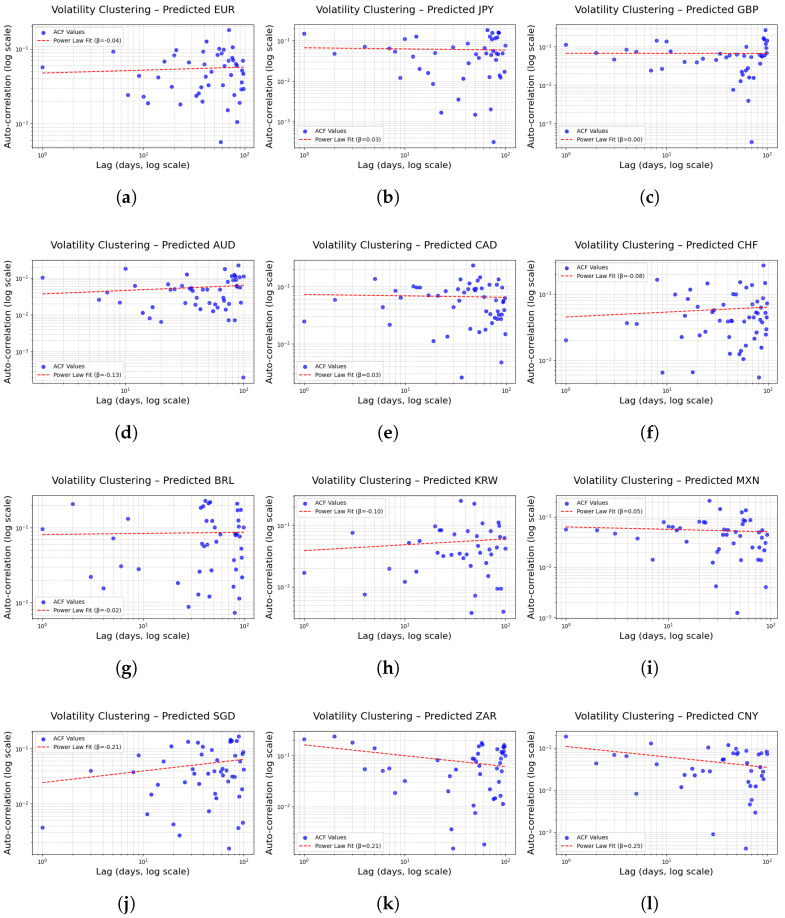
Volatility clustering predicted by FINGAN-BiLSTM. (**a**) EUR, (**b**) JPY, (**c**) GBP, (**d**) AUD, (**e**) CAD, (**f**) CHF, (**g**) BRL, (**h**) KRW, (**i**) MXN, (**j**) SGD, (**k**) ZAR, (**l**) CNY.

Figure 9 and Table 6 present results indicating that the FINGAN-BiLSTM model effectively reproduces the volatility clustering characteristics observed in real FX exchange rate data for most currencies. For instance, in the case of JPY, the clustering index β predicted by the FINGAN-BiLSTM model is 0.0279, reflecting the relatively rapid decay of autocorrelation in absolute returns and appropriately capturing the short-term volatility persistence observed in the data. Moreover, for BRL and MXN, the predicted β values are −0.0150 and 0.0494, respectively, suggesting that despite the presence of outliers and structural volatility inherent in these currencies, the FINGAN-BiLSTM model produces relatively stable clustering characteristics.

In particular, compared to standalone LSTM or BiLSTM models, the FINGAN-based model tends to reproduce the volatility clustering characteristics more realistically. For example, in the case of AUD, the traditional model yielded a β value of −0.1348, indicating a rapid dissipation of volatility, whereas the FINGAN-BiLSTM model reflects a more gradual decay in volatility, capturing the long-term autocorrelation structure of financial time series more accurately. Furthermore, in the case of CHF, the FINGAN-BiLSTM model recorded a β value of −0.0751, indicating better alignment with the observed duration and decay patterns of volatility in the actual data.

However, the predictive performance of the FINGAN-BiLSTM model is not uniformly superior across all FX rates. For instance, in the case of ZAR, although the mean is near zero, as indicated by the basic statistics (Table 1), the high volatility is evident in its Std. dev. (0.0123), and the extreme values (maximum 0.2041, minimum −0.2009) are striking. These characteristics are also clearly observed in the log-return distribution (Figure 1), where the ZAR data exhibit frequent outliers and irregular volatility clustering behavior. Consequently, the FINGAN-BiLSTM model predicted a β value of 0.2121 for ZAR, which deviates from the generally expected clustering pattern (i.e., a mild positive β value). This outcome suggests that the outliers and high volatility in the ZAR data adversely affected the model’s training and generalization.

#### 5.2.3. LeverageEffect

The leverage effect is a stylized fact that quantitatively measures the asymmetric impact of past returns on future volatility, thereby elucidating the asymmetric risk transmission mechanism that occurs during declines in stock prices or exchange rates [51,52].

In this study, the leverage effect for each lag *k* is defined using the predicted logarithmic returns r(t) as follows:

(8) $$L\left(k\right)=\frac{E\left[{r\left(t\right)\;|r\left(t+k\right)|}^{2}-r\left(t\right)\;{\left|r\left(t\right)\right|}^{2}\right]}{{\left(E\left[{\left|r\left(t\right)\right|}^{2}\right]\right)}^{2}}$$

In Equation (8), r(t) denotes the logarithmic return at time *t*, and ${\left|r\left(t\right)\right|}^{2}$ represents the volatility at that time. The numerator, $E\left[{r\left(t\right)\;|r\left(t+k\right)|}^{2}-r\left(t\right)\;{\left|r\left(t\right)\right|}^{2}\right]$, reflects the average difference between future and current volatility at a given lag *k*, while the denominator, ${\left(E\left[{\left|r\left(t\right)\right|}^{2}\right]\right)}^{2}$, normalizes this value by the square of the overall volatility level.

The leverage effect is characterized by the following empirical patterns. First, numerous empirical studies have reported that L(k) assumes negative values, particularly within the lag range 1≤k≤10, indicating that negative past returns significantly increase future volatility in the short term [53,54]. Second, the leverage effect exhibits a gradual decay as the lag increases [11,55].

Given these characteristics, the leverage effect serves as a key metric for quantitatively assessing the asymmetric risk factor that triggers a rapid increase in volatility following declines in asset prices in financial markets.

**Figure 10:**
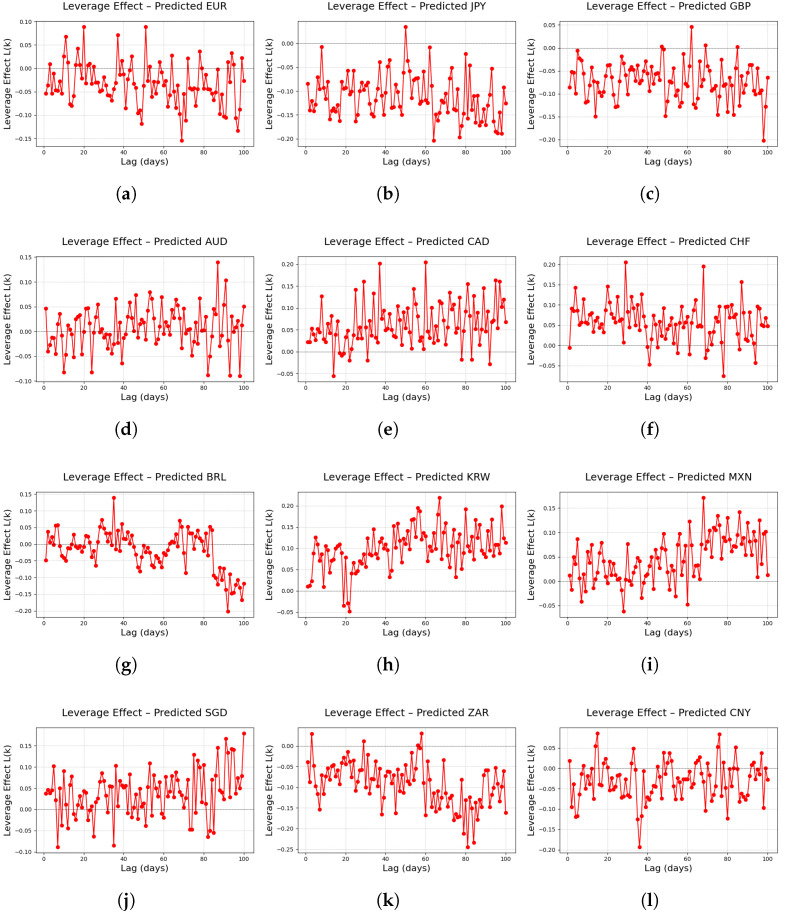
Leverage effect predicted by FINGAN-BiLSTM. (**a**) EUR, (**b**) JPY, (**c**) GBP, (**d**) AUD, (**e**) CAD, (**f**) CHF, (**g**) BRL, (**h**) KRW, (**i**) MXN, (**j**) SGD, (**k**) ZAR, (**l**) CNY.

Figure 10 presents results indicating that the FINGAN-BiLSTM model, overall, successfully reproduces the typical leverage effect observed in financial markets across most FX currencies. In the case of major currencies such as EUR, JPY, and GBP, a clear negative leverage effect is observed at short lags, exhibiting the characteristic pattern of a sharp increase in volatility during market downturns.

However, for major FX currencies such as AUD, CAD, and CHF, instances were observed in which the typical negative leverage effect was not sufficiently manifested. A review of the basic statistics (Table 1) and log-return distributions (Figure 1) for these currencies reveals pronounced characteristics of high volatility, frequent outlier occurrences, and notable asymmetry in their distributions. These properties hinder the model’s ability to fully capture the tail behavior and asymmetric features during training, which may result in a weakened amplification effect of short-term negative returns on future volatility, yielding only a modest negative value or, in some cases, even a positive value. For instance, in the case of CAD, although the conventional LSTM model produced an excessively high tail index due to outlier influence, the FINGAN-BiLSTM model also failed to adequately reflect the true distributional properties and, consequently, did not reproduce the expected negative leverage effect.

For minor FX currencies such as BRL, KRW, MXN, SGD, ZAR, and CNY, external factors such as economic and political instabilities and shifts in international liquidity lead to a more complex and irregular manifestation of the leverage effect. BRL and MXN exhibit a negative leverage effect at short lags; however, as the lag increases, this effect rapidly diminishes or becomes unstable as the lag increases. Similarly, for ZAR and CNY, the high volatility and frequent occurrence of outliers observed in the basic statistics impede the full realization of the typical negative effect.

In summary, while the FINGAN-BiLSTM model successfully reproduces the typical leverage effect in most FX currencies, it falls short in adequately capturing this effect in major currencies characterized by high volatility, frequent outliers, and marked asymmetry (e.g., AUD, CAD, and CHF), as well as in minor currencies that are heavily influenced by external factors (e.g., BRL, KRW, MXN, SGD, ZAR, and CNY).

### 5.3. Risk Measures

In financial time-series analysis, value-at-risk (VaR) and expected shortfall (ES) are widely used metrics for quantifying extreme loss risk. Under a confidence level of α=0.95, the 95% VaR is defined as the (1−α) quantile of the loss distribution. Denoting the daily return by r(t) with cumulative distribution function F(r), the VaR at the 95% level is given by the following:

(9) $${\mathrm{VaR}}_{95}\;=\;\inf\left\{\;x|F(x)\geq 0.05\right\},$$

which implies that losses will not exceed ${\mathrm{VaR}}_{95}$ with 95% probability.

The 95% expected shortfall is the conditional expectation of losses exceeding the 95% VaR and, thus, captures tail risk unaccounted for by VaR alone [56,57]. It is defined as follows:

(10) $${\mathrm{ES}}_{95}\;=\;E\left[r\;|\;r\leq {\mathrm{VaR}}_{95}\right]\;=\;\frac{1}{0.05}\int_{-\infty }^{{\mathrm{VaR}}_{95}}x\;dF\left(x\right),$$

where the denominator 0.05 normalizes the tail probability mass and the integral computes the average of losses in the lower 5% tail.

In this study, we compute ${\mathrm{VaR}}_{95}$ and ${\mathrm{ES}}_{95}$ separately for the synthetic FX log-return series generated by the FINGAN-BiLSTM model and for the corresponding empirical observations using Equations (9) and (10). This comparison allows us to evaluate the model’s capability to replicate extreme-loss behavior, with a particular focus on currency pairs exhibiting frequent outliers and heavy tails.

In Table 7, the comparison between actual and predicted ${\mathrm{VaR}}_{95}$ and ${\mathrm{ES}}_{95}$ demonstrates that the FINGAN-BiLSTM model accurately reproduces tail risk for currencies exhibiting moderate volatility and infrequent extreme events. For example, the AUD has an actual ${\mathrm{VaR}}_{95}$ of 1.1566 versus a predicted value of 1.1609, corresponding to an error rate of only 0.4% (±0.0043). Its ${\mathrm{ES}}_{95}$ is similarly well-captured, with an actual value of 1.5067 compared to 1.5795, indicating that the model reliably learns both mid-range fluctuations and tail losses in a distribution whose kurt is 1.4456. Likewise, for the CAD, the predicted ${\mathrm{ES}}_{95}$ of 1.3498 deviates by merely +2.9% from the actual value of 1.3120, confirming that currencies with skew ranging from –0.058 to 0.083 and kurtosis between 1.4456 and 4.3769 are robustly modeled.

By contrast, performance deteriorates markedly for currencies characterized by frequent extremes or exogenous shocks. The ZAR, with its high kurt (111.1518) and persistent volatility clustering, exhibits substantial underestimation: an actual ${\mathrm{VaR}}_{95}$ of 1.1389 is predicted as 0.8422 (–25.9%), and an actual ${\mathrm{ES}}_{95}$ of 2.6586 is predicted as 1.0270 (–61.4%). Similarly, discontinuities due to policy interventions in the CNY appear to induce opposite biases in the model’s outputs: ${\mathrm{VaR}}_{95}$ is overestimated by +25.6% (actual 0.9546 vs. predicted 1.1999), whereas ${\mathrm{ES}}_{95}$ is underestimated by –21.7% (actual 1.9471 vs. predicted 1.5242). This suggests that the model does not fully capture the impact of exogenous intervention timing on tail-loss magnitude.

In summary, while the FINGAN-BiLSTM model approximates VaR and ES with high fidelity for currencies with moderate tail behavior, it exhibits significant under- or overestimation for those with high extreme-event frequency or policy-driven shocks.

### 5.4. Estimate to APARCH Model

In order to simultaneously capture the asymmetric shock effects and non-stationarity observed in financial time series data, this study further validates model performance by employing the asymmetric power ARCH (APARCH) model. The APARCH model is a generalized specification of conditional variance, defined as follows:

(11) $$\sigma_{t}^{\delta }=\omega +\alpha {\left(|\epsilon_{t-1}|-\gamma \epsilon_{t-1}\right)}^{\delta }+\beta \sigma_{t-1}^{\delta },$$

In Equation (11), $\epsilon_{t}=r\left(t\right)-\mu$ denotes the residual at time *t*, and $\sigma_{t}^{2}$ denotes the corresponding conditional variance. The exponent δ>0 controls the degree of nonlinearity in the variance response. The constant term ω>0 determines the long-run average variance, and the coefficient α≥0 measures the effect of the absolute magnitude of the previous shock on the variance.

Equation (11) relaxes the symmetry assumption of standard GARCH models and generalizes the response to shock magnitude in a power form, thus more accurately capturing tail clustering and the leverage effect observed in financial time series [58,59]. In the empirical analysis of this paper, the parameters (β,γ) in Equation (11) are estimated using quasi-maximum likelihood estimation (QMLE).

The clustering exponent β used in this paper is calculated according to Equation (7), whereas the persistence coefficient β of the APARCH model is defined by Equation (11).

In this study, β is a non-parametric exponent that assumes the ACF of absolute returns at lag *k* decays as a power law, and is estimated by linear fitting in the log–log plot, according to Equation (7).

By contrast, the APARCH model’s β is a parametric coefficient quantifying the autoregressive persistence of the conditional variance $\sigma_{t}^{2}$. In Equation (11), it measures the effect of past variance $\sigma_{t-1}^{\delta }$ on current variance, and is estimated via quasi-maximum likelihood estimation (QMLE).

In conclusion, the β in this study measures the rate of exponential decay of volatility clustering via Equation (7), whereas the APARCH model’s β quantifies volatility persistence in Equation (11).

The leverage–effect statistic L(k) used in this study is computed from Equation (8), whereas the asymmetry parameter γ of the APARCH model is defined by Equation (11).

L(k) is a non-parametric measure obtained from the correlation between past returns r(t) and future volatility ${\left|r\left(t+k\right)\right|}^{2}$ at each lag *k*, using sample covariances and means.

By contrast, the APARCH parameter γ summarizes—using a single constant—the asymmetric adjustment that the sign of the previous shock $\left|\epsilon_{t-1}\right|$ imparts to the current conditional variance $\sigma_{t}^{2}$. It is a parametric quantity estimated as a coefficient in Equation (11) by QMLE.

In Equation (11), γ quantifies the directional difference in volatility responses, capturing the degree of asymmetric risk transfer associated with positive versus negative market shocks.

Table 8 presents a comparison of the persistence coefficient $\beta_{1}$ and the asymmetry coefficient $\gamma_{1}$ of the APARCH model, estimated from the actual time series and from the synthetic time series generated by the FINGAN–BiLSTM model for each currency pair.

For most currency pairs, the absolute difference in $\beta_{1}$ between actual and predicted values is within 0.05, indicating that the proposed model has generally captured the autoregressive persistence of conditional variance accurately. However, for the CHF, the predicted $\beta_{1}=0.9597$ is markedly higher than the actual $\beta_{1}=0.6834$, suggesting that the model assumes nearly permanent persistence in a market where volatility actually decays rapidly. Conversely, for the ZAR, the predicted $\beta_{1}=0.0001$ contrasts sharply with the actual $\beta_{1}=0.6657$, indicating a substantial underestimation of volatility persistence.

The analysis of the asymmetry coefficient $\gamma_{1}$ reveals sign reversals and magnitude discrepancies that vary across currencies. Sign reversals occur only for the AUD and the SGD. The JPY exhibits an overestimation of negative asymmetry, whereas the CAD shows a moderate underestimation of positive asymmetry. For the remaining currencies, the signs of actual and predicted values are consistent, correctly reflecting the direction of the leverage effect; however, the absolute errors in $\gamma_{1}$ remain nontrivial for certain pairs.

In summary, the FINGAN–BiLSTM model reliably reproduces volatility persistence ($\beta_{1}$) for the majority of currency pairs, but it does not yet fully learn the currency-specific leverage effects represented by the asymmetry parameter ($\gamma_{1}$), as evidenced by sign reversals in AUD and SGD and over- or underestimation in JPY and CAD.

## 6. Discussion

We propose a novel FINGAN–BiLSTM model that integrates the conventional FINGAN framework with a bidirectional LSTM structure. This architecture is applied to replicate the complex time-series characteristics and representative stylized facts of FX log-return data. It incorporates BiLSTM layers into the generator, discriminator, and predictor networks, thus enabling the capture of both past and future dependencies and effectively reproducing high volatility, asymmetry, and extreme values observed in actual FX returns. Experimental results show that FINGAN–BiLSTM outperforms conventional LSTM and unidirectional BiLSTM baselines in replicating key stylized facts, confirming its utility for predicting FX return distributions.

The main findings of this study are as follows. First, we propose FINGAN–BiLSTM, a GAN-based time-series model that integrates bidirectional LSTM layers into the original FINGAN framework to address limitations of unidirectional architectures. This model synthesizes realistic financial series via adversarial training while capturing both past and future dependencies, thus reducing information loss inherent in conventional approaches. In addition, FINGAN–BiLSTM preserves complex distributional characteristics through the adversarial interplay of generator and discriminator networks. Furthermore, empirical results show that the bidirectional architecture more accurately reproduces stylized facts and improves distributional predictions of FX log returns compared to standard LSTM and unidirectional BiLSTM baselines, especially for forecasting tasks that require modeling bidirectional temporal dependencies.

Second, our empirical results confirm that FINGAN–BiLSTM outperforms conventional LSTM, BiLSTM, and original FINGAN architectures in reproducing key stylized facts of FX return distributions. Using the Kolmogorov–Smirnov (KS) test, we show that FINGAN–BiLSTM minimizes the maximum divergence between empirical and predicted cumulative distribution functions, indicating that it faithfully reproduces the observed frequency of extreme events and the density of the tail region. It also reproduces volatility clustering by alternating between high and low volatility regimes and captures the leverage effect asymmetry, in which negative returns are followed by short-term volatility increases. These findings demonstrate that FINGAN–BiLSTM overcomes the limitations of existing models and effectively captures the non-stationary, complex dynamics of financial time series.

Third, we demonstrate that FINGAN–BiLSTM integrates adversarial data generation with bidirectional LSTM to forecast FX return distributions beyond simple mean-level predictions. It captures higher-order distributional features and volatility dynamics that unidirectional LSTM models fail to represent. By simultaneously learning past and future dependencies, the model reduces information loss inherent in one-way architectures. This framework demonstrates superior capability in capturing endogenous volatility dynamics, thereby enabling effective modeling of volatility clustering and risk transmission mechanisms under normal market conditions. Furthermore, empirical evaluations confirm that FINGAN–BiLSTM accurately reproduces heavy tails, volatility clustering, and extreme events in FX returns, thereby modeling the complex uncertainties of financial markets.

Based on the results of the empirical tests, we highlight the following key contributions. The proposed FINGAN–BiLSTM model effectively captures complex statistical features of FX returns by integrating BiLSTM with GAN, thus reducing the information loss of unidirectional LSTM architectures. Unlike conventional models that rely solely on past data, BiLSTM learns both forward and backward dependencies. This enables complex representation of nonlinearity, heavy tails, and extreme-event risks during adversarial training [60,61]. Consequently, FINGAN–BiLSTM, such as volatility clustering and leverage effects, while minimizing divergence between empirical and generated distributions. These capabilities enhance extended long-term forecasting performance by providing a more realistic representation of asymmetric and extreme behaviors compared to conventional models based on the normal distribution. Furthermore, they can be effectively applied to practical domains such as risk management, derivative pricing, and portfolio optimization.

In discussing the empirical results, analysis of the KS test across 12 FX assets reveals that EUR, AUD, and GBP exhibited excellent goodness-of-fit, with low KS test values of 0.0586, 0.0613, and 0.0370, respectively, indicating remarkable correspondence between the predicted and empirical distributions. Despite maximum deviations for GBP and EUR occurring in the central percentile regions, the model effectively reproduced the overall distributional shape. AUD also captured moderate-range volatility more precisely than extreme-value dynamics, highlighting the model’s robustness for developed-market currencies. By contrast, CNY exhibited a relatively higher KS test owing to frequent structural shifts driven by aggressive central bank interventions and policy adjustments, and ZAR’s performance was similarly impaired by its extreme values and pronounced volatility; in both cases, the model trained primarily on historical data was unable to fully accommodate abrupt distributional changes [37,38].

Regarding outliers, the KS test statistic for the BRL series was 0.0894 prior to the removal of outliers. After excluding 27 outliers (approximately 1.5% of the sample) using an interquartile range (IQR)–based filter, the KS test decreased to 0.0488, indicating that the distributional discrepancy was substantially reduced. In contrast, the ZAR series exhibited a KS test of 0.1545 both before and after removal of 12 outliers (approximately 0.8% of the sample), suggesting that filtering alone did not resolve the mismatch. These results indicate that removing more than 1% of observations can markedly improve model fit by attenuating extreme-value effects. Conversely, the exclusion of fewer than 1% of data points has a negligible impact. Moreover, owing to the inherently high volatility of the ZAR series, its empirical distribution remains unrepeatable even after outlier exclusion.

Furthermore, the heavy-tailed analysis revealed that the FINGAN-BiLSTM model generally reproduced tails similar to those of the actual data. For instance, assets such as EUR, JPY, and GBP yielded predicted tail indices (α in Equation (5)) that fell within the typical range (approximately 3 to 5) observed in financial time series, thereby realistically reflecting the probability of extreme events. In contrast, while a unidirectional LSTM produced an excessively high tail index for CAD, the FINGAN-BiLSTM model effectively corrected this value to a realistic level. Although MXN exhibited some instability in replicating heavy-tailed characteristics due to the influence of outliers, overall, the model successfully captured the heavy-tailed property.

In the volatility clustering analysis, assets with pronounced volatility, such as JPY, BRL, and MXN, showed that the FINGAN-BiLSTM model effectively captured the alternating pattern of high and low volatility periods. In particular, for JPY, the predicted clustering index (β in Equation (7)) accurately reflected the short-term persistence of volatility observed in the data, and for AUD and CHF, the model reproduced a more gradual clustering effect compared to conventional models, thereby improving the modeling of long-term temporal dependencies. However, for assets like ZAR, which exhibit extreme values and high volatility, the reproduction of volatility clustering was unstable, resulting in clustering indices that deviated from the expected range.

The leverage effect analysis demonstrated that, for major assets such as EUR, JPY, and GBP, a clear negative leverage effect was observed, indicating that negative returns were followed by an increase in short-term volatility. This result indicates that the FINGAN-BiLSTM model effectively captures the asymmetric leverage effect of each FX series. Conversely, for assets such as AUD, CAD, and CHF, which display high volatility and frequent extreme values, the model occasionally failed to fully reproduce the asymmetric nature of the leverage effect. This suggests that during the training process, the extreme features in the original data distribution may not have been entirely captured, leading to discrepancies between the predicted and actual outcomes. Additionally, for minor assets like CNY and ZAR that are heavily influenced by external factors, the leverage effect was found to be unstable, indicating that further development of models incorporating external variables is warranted.

Overall, the FINGAN–BiLSTM model demonstrated robust performance in reproducing key stylized facts of FX returns, including low KS test values, realistic heavy-tailed properties, effective volatility clustering, and discernible leverage effects for assets such as CAD, EUR, JPY, and GBP. However, its performance was less robust for assets subject to significant external shocks or frequent extreme values, notably CNY and ZAR. These findings underscore the need for FX return model tuning and suggest directions for future research to more precisely capture the extreme dynamics of financial time-series data.

## 7. Concluding Remarks

Our results demonstrate that FINGAN–BiLSTM accurately replicates the complex dynamics of financial time series by reproducing heavy-tailed behavior, volatility clustering, and leverage effects—features often disregarded by traditional models based on the normal distribution. Moreover, the proposed model provides a data-driven framework for quantifying 99% tail losses and directional spillovers between asset classes. In practical applications, FINGAN–BiLSTM improves market risk assessment through precise VaR estimation, enhances derivative pricing via simulated return paths, and supports portfolio optimization by quantifying asymmetric risk transmission. Overall, this study validates the academic and practical viability of deep learning–based forecasting methods for financial time series and opens new directions in financial data analysis, risk management, and decision-making frameworks.

Nonetheless, the dependence of the FINGAN-BiLSTM model on historical data limits its effectiveness in capturing the dynamics of asset markets like CNY and ZAR, where sudden structural shifts frequently occur due to significant central bank interventions and policy changes. Moreover, for assets characterized by frequent extreme events, the model fails to consistently capture the tail distribution, resulting in discrepancies between the predicted and empirical distributions. When evaluated against synthetic time series benchmarks based on GARCH-family models, certain currency pairs demonstrated a failure to fully reflect the actual volatility structure or tail characteristics; this shortcoming is attributable to GARCH models’ focus on traditional moment-based conditional variances, which inadequately account for extreme event frequency, long-range dependencies, and asymmetric effects [62,63,64].

To address these limitations, we propose several directions for future research. First, to overcome the model’s current limitation in capturing the tail behavior of return distributions, we propose the development of enhanced modeling frameworks that integrate exogenous variables, such as macroeconomic conditions, geopolitical risks, and policy interventions, capable of accommodating regime shifts under extreme market scenarios. Furthermore, to reduce biases introduced by extreme observations, future research can incorporate asset-specific calibration strategies, employ sophisticated normalization methods, and design customized loss functions aligned with the distributional characteristics of financial assets.

Second, we suggest conducting comprehensive empirical validations across diverse asset classes and extended time horizons to reinforce the robustness of our findings. Furthermore, to enhance risk management and portfolio optimization, we encourage further investigation into the estimation of VaR and ES using conditional return distribution models.

Finally, as the present study is limited to a univariate conditional distribution framework, future research can extend the model to multivariate conditional settings. This extension would enable the modeling of cross-asset return dynamics under structural changes and thus improve the model’s applicability to portfolio-level risk management and asset allocation strategies.

## Figures and Tables

**Table 1:** The Jarque–Bera statistic tests the null hypothesis of normality for the sample returns. ^‡^ indicates a rejection of the null hypothesis at the 1% significance level. Std. dev. skew. kurt. J.-B. mean standard deviation, skewness, kurtosis, and Jarque–Bera statistics, respectively. ADF and PP are the augmented Dickey–Fuller and Phillips–Perron, respectively. ^‡^ indicates a rejection of the null hypothesis at the 1% significance level.

FX	Mean	Max.	Min.	Std. dev.	Skew.	Kurt.	J.-B.	ADF	PP
EUR	−0.0001	0.0182	−0.0281	0.0047	−0.1477	1.9901	217.11 ^‡^	−35.08 ^‡^	−35.10 ^‡^
JPY	−0.0003	0.0380	−0.0267	0.0058	0.3822	3.9391	865.66 ^‡^	−35.57 ^‡^	−35.61 ^‡^
GBP	0.0001	0.0303	−0.0423	0.0058	−0.3988	6.0108	1978.02 ^‡^	−16.29 ^‡^	−34.81 ^‡^
AUD	−0.0001	0.0285	−0.0322	0.0068	−0.0581	1.4456	112.60 ^‡^	−35.71 ^‡^	−35.72 ^‡^
CAD	−0.0001	0.0319	−0.0297	0.0044	−0.0167	4.3769	1030.01 ^‡^	−18.02 ^‡^	−37.27 ^‡^
CHF	0.0001	0.0284	−0.0226	0.0048	0.2965	2.4943	352.98 ^‡^	−34.37 ^‡^	−34.38 ^‡^
BRL	−0.0003	0.0907	−0.0863	0.0111	0.0834	7.6854	3179.91 ^‡^	−40.02 ^‡^	−40.26 ^‡^
KRW	−0.0002	0.0350	−0.0205	0.0057	0.3813	2.4597	356.24 ^‡^	−15.91 ^‡^	−39.60 ^‡^
MXN	−0.0001	0.0326	−0.0608	0.0084	−0.8951	4.7591	1391.47 ^‡^	−35.06 ^‡^	−35.10 ^‡^
SGD	0.0001	0.0129	−0.0150	0.0029	−0.0486	1.7432	163.37 ^‡^	−36.18 ^‡^	−36.22 ^‡^
ZAR	−0.0002	0.2041	−0.2009	0.0123	0.0616	111.1518	665,572.10 ^‡^	−16.26 ^‡^	−47.53 ^‡^
CNY	0.0001	0.0216	−0.0212	0.0036	0.3246	7.7546	3258.75 ^‡^	−6.66 ^‡^	−43.98 ^‡^

**Table 2:** Summary of key hyper-parameters and training settings.

Component	Hyperparameter	Value
Data	Sequence length *L*	15
	Log-return scaling	mean = 0, var = 1
	Positive-return normalization	divide by std
	Batch size	128
Generator	Noise dimension	64
	BiLSTM layers	2 × 128 units, dropout 0.10
	Layer normalization	yes
	Gaussian noise std	0.10
Discriminator	BiLSTM layer	1 × 32 units, dropout 0.005
	Dense + dropout	tanh, Drop(0.01)
	Flatten → Linear	yes
Predictor	BiLSTM layer	1 × 128 units, dropout 0.001
	Dense + dropout	tanh, Drop(0.001)
	Flatten → Linear	yes
Training	Epochs	1000
	Optimizer	Adam
	Learning rates	*G*: 5 $\times \;10^{-4}$, *D*: 2 $\times \;10^{-4}$, *P*: 5 $\times \;10^{-4}$
	Betas	$\beta_{1}=0.0,\;\beta_{2}=0.9$ (AMSGrad for *G*)
	Gradient clipping	norm 1.0
Regularization	Auxiliary loss weight	0.01
	Drift regularization	1 $\times \;10^{-5}$
	Gradient-penalty weight	1.0

**Table 3:** Kolmogorov–Smirnov Statistics for individual tickers analyzed by each model.

FX	LSTM	BiLSTM	FINGAN-LSTM	FINGAN-BiLSTM
EUR	0.0772	0.1626	0.0853	0.0610
JPY	0.3699	0.1341	0.1138	0.1179
GBP	0.1016	0.1219	0.1178	0.0407
AUD	0.1056	0.1341	0.0853	0.0650
CAD	0.5365	0.1178	0.0934	0.0894
CHF	0.0975	0.1463	0.0853	0.0772
BRL	0.0772	0.1544	0.0975	0.0894
KRW	0.1747	0.1666	0.1219	0.1260
MXN	0.5406	0.1788	0.1097	0.1585
SGD	0.1666	0.1626	0.1097	0.0651
ZAR	0.4105	0.1341	0.0813	0.1545
CNY	0.1097	0.0975	0.1260	0.2358

**Table 4:** Test α and β values for selected FX pairs. Here, α is the tail exponent, and β is the volatility decay exponent.

FX	EUR	JPY	GBP	AUD	CAD	CHF
α	3.5388	3.1731	5.2031	3.0902	5.5990	4.8114
β	−0.1134	0.1646	−0.0420	0.0771	0.0922	−0.1342
FX	BRL	KRW	MXN	SGD	ZAR	CNY
α	3.0943	3.3335	2.5402	3.4561	2.9068	3.1112
β	0.2985	0.0813	0.4423	0.1416	3.4440	0.6278

**Table 5:** Predicted heavy-tailed parameters (α) of FX log-return distributions by FINGAN-BiLSTM.

FX	LSTM	BiLSTM	FINGAN-LSTM	FINGAN-BiLSTM
EUR	6.7527	4.1954	3.6676	4.4033
JPY	3.4936	3.9288	2.7452	4.0506
GBP	3.5954	16.1255	4.4847	4.0041
AUD	3.0550	7.5813	3.4736	3.5274
CAD	2.5100	6.9978	4.1686	3.4677
CHF	3.4790	3.0392	4.5360	4.5724
BRL	2.7948	2.8501	5.2073	3.3174
KRW	5.9826	4.8150	3.6240	4.5802
MXN	4.2139	2.7520	5.6271	3.5098
SGD	3.6452	5.6661	3.6266	3.6820
ZAR	2.6344	4.0989	2.4277	2.9069
CNY	2.8036	2.6435	2.0566	3.8045

**Table 6:** Predicted power-law exponents (β) of volatility clustering in fx exchange rates by FINGAN-BiLSTM.

FX	LSTM	BiLSTM	FINGAN-LSTM	FINGAN-BiLSTM
EUR	0.0952	0.0249	0.0545	−0.0395
JPY	−0.0834	0.2954	0.3330	0.0279
GBP	0.0037	−0.1873	0.0434	0.0018
AUD	0.1626	−0.0497	0.2103	−0.1348
CAD	−0.1970	0.1445	−0.0071	0.0252
CHF	0.0203	0.0706	0.1464	−0.0751
BRL	−0.0518	0.0292	0.2250	−0.0150
KRW	0.0519	−0.1307	0.1174	−0.0951
MXN	0.0668	0.4716	0.3997	0.0494
SGD	−0.0726	−0.1077	0.5458	−0.2113
ZAR	0.0529	−0.0556	0.4518	0.2121
CNY	0.5594	0.7855	0.3353	0.2482

**Table 7:** Comparison of empirical and FINGAN-BiLSTM synthesized time series in terms of VaR_95_ and ES_95_.

	Actual		Predicted	
**Currency**	**VaR_95_**	**ES_95_**	**VaR_95_**	**ES_95_**
EUR	1.1497	1.4718	1.1578	1.4097
JPY	2.0495	2.8218	1.7404	2.0546
GBP	1.0436	1.2040	0.9399	1.4654
AUD	1.1566	1.5067	1.1609	1.5795
CAD	0.9381	1.3120	1.1044	1.3498
CHF	1.2779	1.8245	1.0258	1.4625
BRL	1.1081	2.0750	1.1514	1.4436
KRW	1.3750	1.9727	1.0407	1.4344
MXN	1.4050	1.9031	1.0891	1.3437
SGD	1.4940	2.0355	1.2158	1.4923
ZAR	1.1389	2.6586	0.8422	1.0270
CNY	0.9546	1.9471	1.1999	1.5242

**Table 8:** Comparison of empirical and FINGAN-BiLSTM synthesized time series in terms of APARCH-based $\beta_{1}$ and $\gamma_{1}$.

	Actual		Predicted	
**Currency**	$\beta_{1}$	$\gamma_{1}$	$\beta_{1}$	$\gamma_{1}$
EUR	0.7745	0.9990	0.8289	0.9976
JPY	0.9867	−0.5796	0.9689	−0.9990
GBP	0.9852	0.9999	0.9818	0.9956
AUD	0.9990	−0.9998	0.8314	0.9996
CAD	0.9674	0.9999	0.9204	0.9959
CHF	0.6834	1.0000	0.9597	0.9996
BRL	0.8883	1.0000	0.9990	0.5810
KRW	0.9721	0.4458	0.9781	0.3927
MXN	0.8076	0.9720	0.8067	0.9720
SGD	0.9556	−0.3758	0.9578	0.9944
ZAR	0.6657	0.4682	0.0001	0.4704
CNY	0.3672	0.6183	0.4012	0.9308

## Data Availability

The data used to support the findings of this study are available from the corresponding author upon request.

## Abbreviations

The following abbreviations are used in this manuscript:

ACF	Autocorrelation Coefficient Function
ADF	Augmented Dickey–Fuller
AE	Autoencoder
AI	Artificial Intelligence
ANN	Artificial Neural Network
APARCH	Asymmetric Power ARCH
ARCH	Autoregressive Conditional Heteroskedasticity
ARIMA	Autoregressive Integrated Moving Average
ARMA	Autoregressive Moving Average
AUC	Area Under the ROC Curve
AUD	Australian Dollar
BiGAN	Bidirectional GAN
BiLSTM	Bidirectional LSTM
BPVIX	British Pound Volatility Index
BRL	Brazilian Real
CAD	Canadian Dollar
CCDF	Complementary Cumulative Distribution Function
CDF	Cumulative Distribution Function
CHF	Swiss Franc
CNY	Chinese Yuan
CRM	Customer Relationship Management
ECDF	Empirical Cumulative Distribution Functions
EDF	Empirical Distribution Function
ES	Expected Shortfall
EUR	Euro
EUVIX	Euro Volatility Index
FINGAN	Financial Generative Adversarial Network
FINGAN-BiLSTM	Financial Generative Adversarial Network–Bidirectional Long-Short Term Memory
FX	Foreign exchange
FXVIX	FX Volatility Index
GAN	Generative Adversarial Network
GP	Gaussian Processes
GARCH	Generalized Autoregressive Conditional Heteroscedasticity
GDP	Gross Domestic Product
GRU	Gated Recurrent Unit
HMM	Hidden Markov Model
i.i.d.	Independent and Identically Distributed
IQR	Interquartile Range
J.-B.	Jarque–Bera
JPY	Japanese Yen
JYVIX	Japanese Yen Volatility Index
KS	Kolmogorov–Smirnov
KS_Stat	Kolmogorov–Smirnov Statistic
KOSPI 200	Korea Composite Stock Price Index 200
Kurt	Kurtosis
KRW	Korean Won
LASSO	Least Absolute Shrinkage and Selection Operator
LSTM	Long-Short Term Memory
LSGAN	L2 loss-based GAN
MAE	Mean Absolute Error
MAPE	Mean Absolute Percentage Error
MCMC	Markov Chain Monte Carlo
ML	Machine Learning
MLP	Multi-Layer Perceptron
MSE	Mean Squared Error
MXN	Mexican Peso
PDF	Probability Density Function
PP	Phillips–Perron
QMLE	Quasi-Maximum Likelihood Estimation
QQ plot	Quantile–Quantile plot
RMSE	Root Mean Squared Error
RNN	Recurrent Neural Network
S&P 500	Standard & Poor’s 500 Index
SHCOMP	Shanghai Stock Exchange
Skew	Skewness
SGD	Singapore Dollar
Std. dev.	Standard Deviation
SVM	Support Vector Machine
TSX	Toronto Stock Exchange
VAE	Variational Autoencoder
VaR	Value-at-Risk
